# New Triphenylphosphonium Salts of Spiropyrans: Synthesis and Photochromic Properties

**DOI:** 10.3390/molecules29020368

**Published:** 2024-01-11

**Authors:** Artur A. Khuzin, Dim I. Galimov, Liliya L. Khuzina, Adis A. Tukhbatullin

**Affiliations:** Institute of Petrochemistry and Catalysis, Ufa Federal Research Center of the Russian Academy of Sciences, 141 Oktyabrya Prospect, 450075 Ufa, Russia; galimovdi@mail.ru (D.I.G.); khuzinall@mail.ru (L.L.K.); adiska0501@gmail.com (A.A.T.)

**Keywords:** spiropyran, merocyanine, photochromism, spectral–kinetic properties, luminescent properties, photodegradation, triphenylphosphonium salts, triphenylphosphonium cation

## Abstract

The most important area of modern pharmacology is the targeted delivery of drugs, and one of the most promising classes of chemical compounds for creating drugs of this kind are the photochromic spiropyrans, capable of light-controlled biological activity. This work is devoted to the synthesis and study of the photochromic properties of new triphenylphosphonium salts of spiropyrans. It was found that all the synthesized cationic spiropyrans have high photosensitivity, increased resistance to photodegradation and the ability for photoluminescence.

## 1. Introduction

Reversible molecular rearrangements play an important role in the study of many biological, chemical and physicochemical processes, wherein photochromic transformations are of great interest—reversible changes in a molecule between two nonequivalent forms with different absorption spectra, induced by external influences. The development of new organic photochromic systems and materials based on them is one of the areas of modern organic synthesis.

Among the most studied classes of organic photochromic structures are spirocyclic compounds, such as spiropyrans, which are capable of switching between the usually colorless cyclic and colored merocyanine forms when exposed to external stimuli such as light [1,2,3,4], temperature [4,5], pressure [6], environmental polarity [2,7], pH [2,8,9], mechanical action [2,10], electric or magnetic field [2,3], which allows their use in memory elements [2,3,11,12], as optoelectronic devices [13,14], sensors [1,2,15,16,17,18], light-responsive materials in biology [2,19,20,21], etc.

Recently, interdisciplinary research aimed at studying controlled drug delivery systems has become increasingly popular. This is due to the fact that the targeted delivery of the drug to the affected area and in controlled quantities can reduce unwanted side effects caused by drug toxicity. Photochromic spiropyrans are promising agents for the creation of drugs of this kind, with a high selectivity and a high induced therapeutic effect, due to the ability to exist in two isomeric forms with different physicochemical properties. At the same time, in the global literature, there is only one work describing, using one example of water-soluble spiropyran, photoswitchable cytotoxicity towards Hek293 cancer cells [20]. This approach will make it possible to hope that the closed form will not cause a toxic effect and will easily pass through the cell membrane, while the open form, on the contrary, will lead to an induced cytotoxic effect.

It is known that mitochondrial oxidative phosphorylation is the basis of cell life and death. Therefore, the introduction into the structure of spiropyrans of the triphenylphosphonium cation, which has a known ability to quickly penetrate through lipid membranes [22,23,24,25,26,27,28,29], and concentrate mainly in mitochondria, helps to manipulate mitochondrial functions, causing controlled apoptosis [30,31], which will not only enhance the cytotoxic effect, but also use it as a vector for the fast and targeted delivery of the active substance to affected target cells. Moreover, the presence of a positive photochromic effect and luminescent properties will contribute to the additional visualization of intracellular processes.

Previously [32], we synthesized ammonium salts of spiropyrans, which were subsequently tested on their cytotoxic activity against various cancer cell lines, including MCF7, A431, BT474, and HF (unpublished data). The results obtained to date cannot be called impressive. For example, when a culture of A431 human epidermoid carcinoma cells was exposed to ammonium salts, the proportion of living cells was in the range of 82 to 96%. Upon subsequent exposure to UV light, the proportion of living cells ranged from 66 to 91%.

In continuation of these studies, in order to develop the synthesis of new previously unstudied photochromic compounds that are promising for the creation of mitochondria-targeted antitumor drugs, in this work, we synthesized salts of indoline spiropyrans containing a lipophilic triphenylphosphonium cation in their structure, and also their photochromic and luminescent properties were studied.

## 2. Results

### 2.1. Synthesis and Identification

**Scheme 1:**
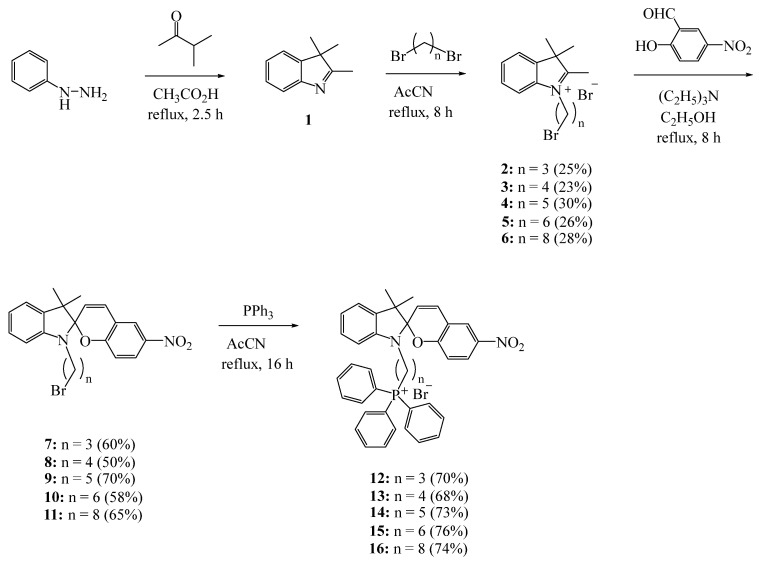
Synthesis of compounds **12**–**16**.

The synthesis of indoline spiropyrans was carried out using methods described in the literature [32,33], according to Scheme 1:

All synthesized triphenylphosphonium salts **12**–**16** were isolated from the reaction mixture using column chromatography. The structures of compounds **12**–**16** were determined using NMR (^1^H, ^13^C and ^31^P) spectroscopy, as well as HRMS high-resolution mass spectrometry (see Supplementary Materials).

**Figure 1:**
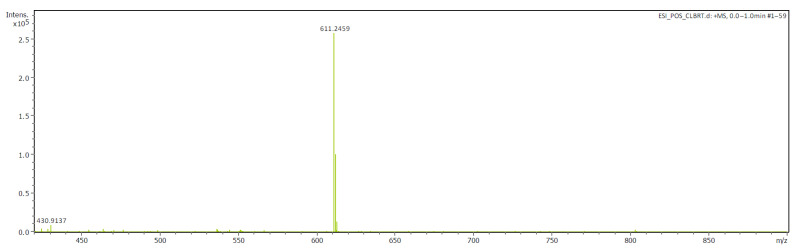
ESI–HRMS spectra of compound **12**.

The structure of the synthesized compounds **12**–**16** was also confirmed by high-resolution mass spectrometry. For example, the HRMS spectrum of compound **12** exhibits an intense peak of a molecular ion with the *m*/*z* C_39_H_36_N_2_O_3_P [M–Br^−^] = 611.2459 (calculated: for MBr = 690.1646 *m*/*z*; for [M–Br^−^] = 611.2458 *m*/*z*) (Figure 1).

The spectra of compounds **13**–**16** are presented in the Supplementary Materials.

### 2.2. UV–Vis and Photoluminescent Studies

Taking into account the sensitivity of spiropyrans to a wide range of external stimuli [1,2,3,4,5,9,34,35,36,37,38,39,40], we studied the photoinduced transformations of synthesized salts **12**–**16** in ethanol using absorption and luminescence spectroscopy. The choice of the latter as a solvent was due to the excellent solubility of the synthesized compounds in ethanol, as well as plans to conduct tests for biological activity in aqueous-alcoholic solutions.

The measurement of the absorption spectra of salts **12**–**16** without and with irradiation with ultraviolet (UV) light showed that all the synthesized compounds have positive photochromism. The results of the measurements of the main spectral and kinetic characteristics of compounds **12**–**16** are summarized in Table 1.

**Figure 2:**
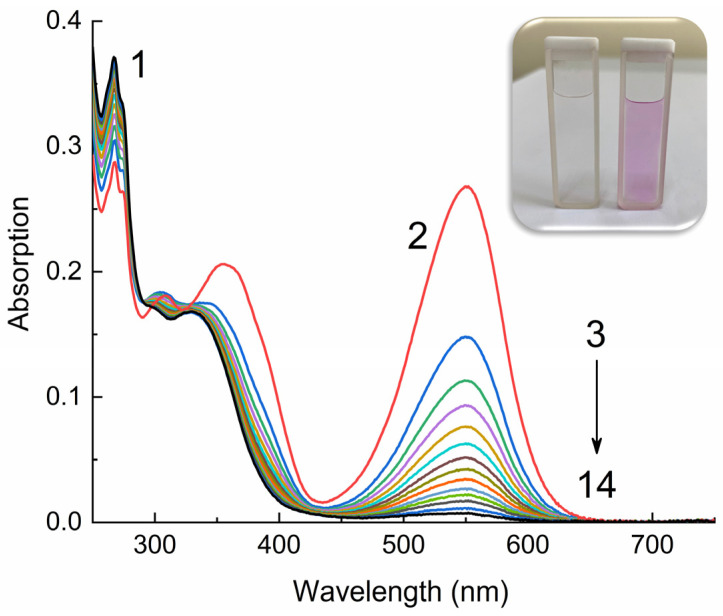
Absorption spectra of compound **12** in ethanol in closed spirocyclic (1) and open merocyanine (2–14) forms, measured before irradiation (1), upon irradiation with UV light through a UFS-1 filter (Elektrosteklo LLC, Moscow, Russia) (2) and upon dark bleaching (3–14). C = 10^−4^ M, l = 0.1 cm, spectrophotometer Agilent Cary-60 (Agilent, Santa Clara, CA, USA). The inset shows a photograph of a solution of **12** in ethanol before (left) and after irradiation (right).

Figure 2, using **12** as an example, shows the typical absorption spectra of spiropyrans **12**–**16** in ethanol without and with UV irradiation. The absorption of spiropyran molecules **12**–**16** in the closed cyclic form is characterized by intense bands in the short wavelength region of the spectrum at 224–225, 267, and 335–339 nm (Figure 2, curve 1).

As can be seen from the data in Table 1, the position of the absorption bands of the spiropyrans slightly (Δ_max_ = 4 nm) shifts to longer wavelengths with an increase in the number of methylene groups in the spacer. When solutions **12**–**16** are irradiated with UV light, the intensity of the absorption bands at 224–225 and 267–275 nm slightly decreases and a new group of bands appears at 309–310, 361–365 and 542–551 nm (Figure 2, curves 3–14).

**Scheme 2:**
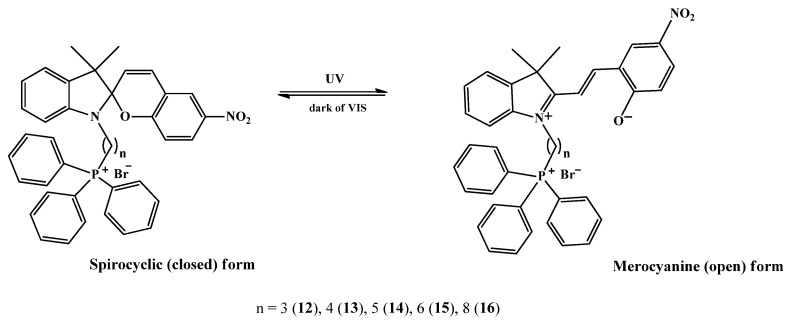
Spiropyran (SP) closed-ring structure and stimuli-induced open-ring isomers (MC).

This change observed in the absorption spectra clearly indicate that under the influence of activating UV radiation, photochromic transformations occur; the pyran ring of molecules **12**–**16** opens and the merocyanine form of the compounds is formed (Scheme 2). In this case, the initially colorless solution instantly acquires a pink color (Figure 2, inset), due to the absorption of merocyanines in the green region of the visible spectrum. The position of this long wavelength maximum (542–551 nm) in the absorption spectrum of merocyanines, as in the case of the closed forms of spiropyrans, depends on the length of the spacer. When going from compound **12** to **14**, a small hypsochromic shift is observed (Δ_max_ = 9 nm). Considering that the absorption bands of spiropyrans, both in open and closed forms, are caused by singlet transitions S_0_ → S*_n_* between n-π* and π-π* orbitals localized on the indole and pyran fragments, these shifts of absorption bands with increasing spacer length can be associated with the effect of the triphenylphosphonium cation on the electron density of the indole fragment.

**Figure 3:**
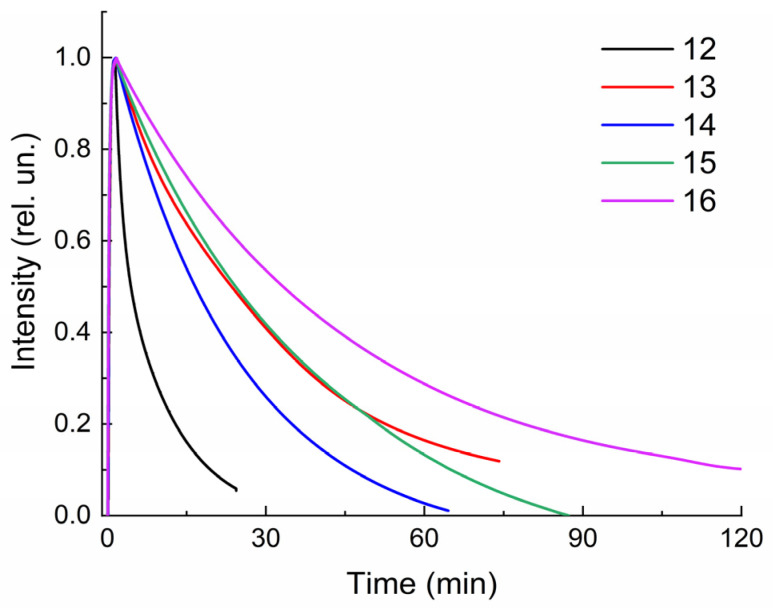
Normalized kinetic curves of dark bleaching of solutions **12**–**16** in ethanol. C = 10^−4^ M, l = 0.1 cm, spectrophotometer Agilent Cary-60.

However, these changes are reversible; therefore, in the dark (dark relaxation, dark bleaching) or upon irradiation with visible light (photorelaxation, photobleaching), molecules **12**–**16** cyclize again, forming the original closed form (Scheme 2). A comparison of the kinetic curves of the dark relaxation process of spiropyrans **12**–**16** (Figure 3) shows that compound **12** has the highest rate of dark bleaching, and compound **16**, on the contrary, has the lowest.

**Figure 4:**
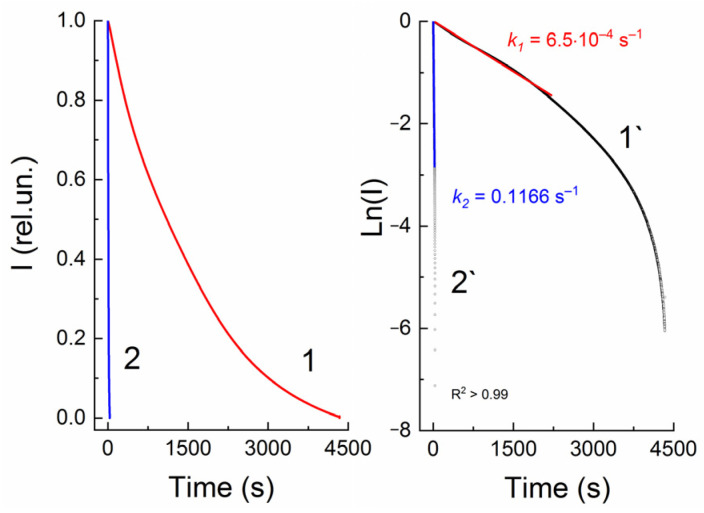
Normalized kinetic curves of dark bleaching (1) and photobleaching of spiropyran **13**, measured at a wavelength of 547 nm, without (1) and with visible light irradiation (2) through an SZS-9 light filter (Elektrosteklo LLC, Moscow, Russia). 1` and 2`—linear anamorphoses of kinetic curves 1 and 2 in coordinates Ln(I)–t(s). C = 10^−4^ M, l = 0.1 cm, spectrophotometer Agilent Cary-60.

This observation is confirmed by a quantitative assessment of the rate constants of spontaneous dark bleaching k_1_ (Table 1), which are obtained from the slope of linear dependences when constructing kinetic curves in the Ln(I)–t(c) coordinates (Figure 4). For all studied compounds **12**–**16**, the initial section of anamorphoses has a linear appearance, i.e., dark relaxation is described by a first-order reaction equation.

The kinetics of death of merocyanines upon irradiation with visible light (SZS-9 light filter, maximum transmission wavelength 490 nm) is also described by a first-order equation for all spiropyrans **12**–**16** (Figure 4). Table 1 shows that the rate constants for photobleaching of compounds **12**–**16** are three orders of magnitude greater than the rate constant for dark bleaching.

**Figure 5:**
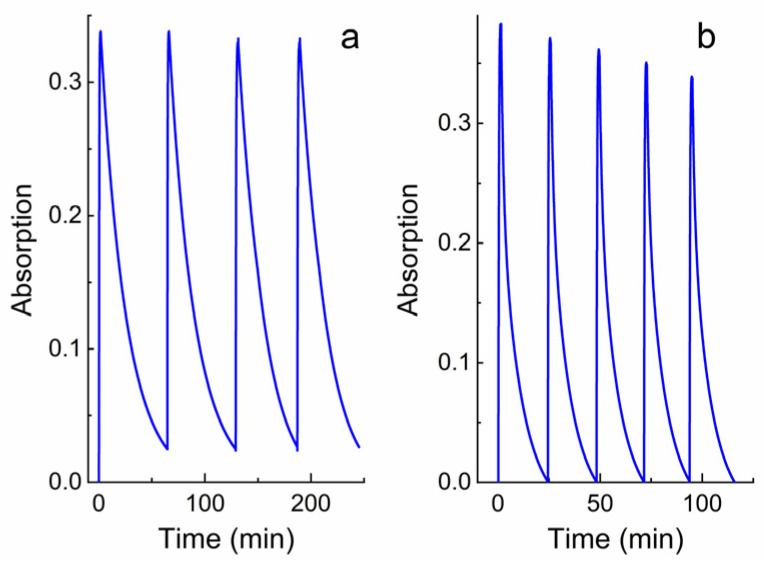
Kinetics of alternating photocoloration of solutions **12** (**a**) and **14** (**b**) in ethanol under the influence of UV light (through a UFS-1 filter (Elektrosteklo LLC, Moscow, Russia)) and dark bleaching. Kinetic curves were obtained spectrophotometrically at wavelengths of 551 and 542 nm, respectively. C = 10^−4^ M, l = 0.1 cm, spectrophotometer Agilent Cary-60.

**Figure 6:**
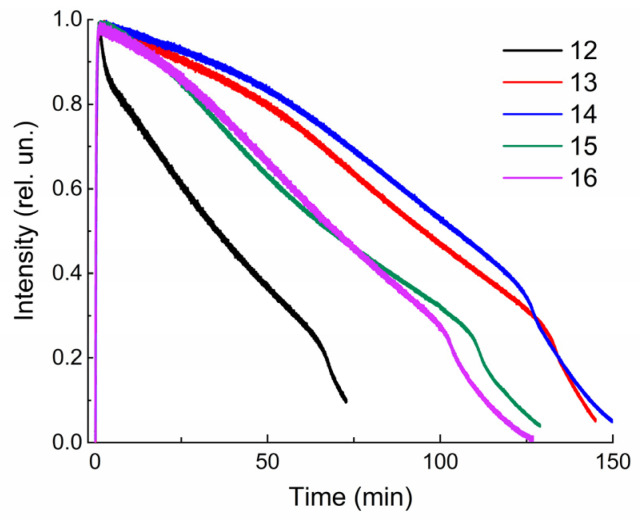
Normalized kinetic curves of photodegradation of solutions **12**–**16** in ethanol under the influence of UV light through a UFS-1 (Elektrosteklo LLC, Moscow, Russia) light filter, measured at the corresponding wavelengths (see Table 1). C = 10^−4^ M, l = 0.1 cm, spectrophotometer Agilent Cary-60.

The measurements of the kinetic curves of the alternating photocoloration and dark bleaching of solutions **12**–**16** showed that the switching cycle can be repeated many times (Figure 5). However, after each «irradiation–relaxation» cycle, a decrease in the optical density of compounds **12**–**16** is observed, which may be due to their photodestruction. The efficiency of the photodegradation reaction measured from the absorption spectra (Figure 6, Table 1) confirms this assumption. Based on Table 1, it can be stated that with the constant irradiation of the solutions of spiropyrans **12**–**16** with UV light, the optical density at the maximum of the absorption band of the merocyanine form decreases. Moreover, for these connections, this time ranges from 32 to 99 min. Moreover, spiropyran **12** is characterized by the most pronounced photodegradation. Although the photosensitivity, determined by the ∆D^max^/D^max^ ratio, is almost the same for all spiropyrans, **12**–**16,** studied (Table 1). The detected difference in the measured kinetic characteristics of compounds **12**–**16** confirms our assumption that in the case of a short spacer length (*n* = 3 for **12**), the triphenylphosphonium cation is able to influence the spiropyran molecule. A detailed explanation of the influence of the spacer length on the spectral–kinetic characteristics of the spiropyrans under study requires special experiments and theoretical calculations, and will be the subject of our future research.

It is known that photochromic compounds capable of luminescence are of great interest because they can be used as luminescent molecular switches [2,3,4]. In addition, luminescence analysis is one of the main methods for studying the state of matter in biological systems, and the study of luminescence spectra is a necessary step in the study of complex photobiological systems. In this regard, we studied the spectral and luminescent properties of the synthesized triphenylphosphonium salts **12**–**16** in ethanol at room temperature (25 °C).

**Figure 7:**
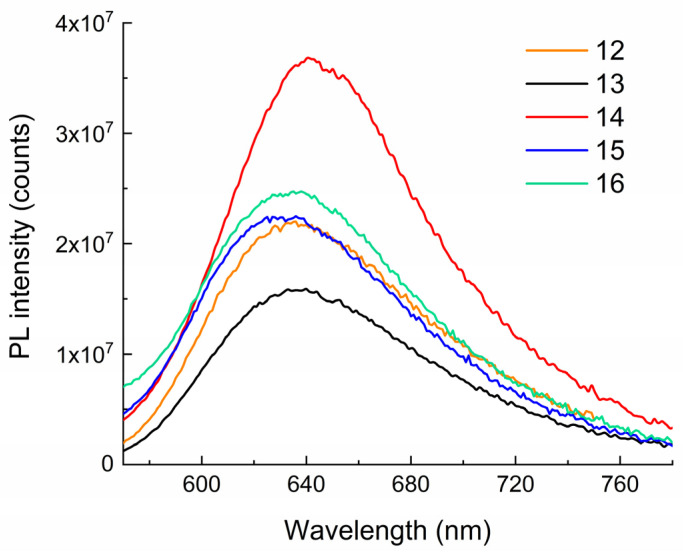
The PL spectra of compounds **12**–**16** in ethanol. λ_exc_ = 550 nm. T = 298 K. C = 10^−4^ M, l = 0.1 cm, Horiba Jobin Yvon Fluorolog-3 spectrofluorometer (Horiba, Kyoto, Japan).

As expected, no photoluminescence (PL) was detected for the spirocyclic forms of the synthesized compounds **12**–**16**. These data are in good agreement with the known literature data [4,7] and our previous experiments on studying the PL of spiropyrans of various chemical structures [32,40]. However, upon irradiation of solutions of **12**–**16** in ethanol, PL in the red region of the visible spectrum with a maximum at 636–640 nm was detected (Figure 7). As can be seen from this figure, the position of the maxima in the spectra and the PL intensity slightly depend on the number of methylene groups in the spiropyran spacer.

## 3. Materials and Methods

The ^1^H, ^13^C and ^31^P NMR spectra were run on a Bruker Avance-500 spectrometer (Bruker, Billerica, MA, USA) at 500.17 and 125.78 MHz or on a Bruker Avance-400.13 spectrometer (Bruker, Billerica, MA, USA) at 400.17 and 100.62 MHz, respectively. CDCl_3_ was used as a solvent. High-resolution mass spectrometry (HRMS) was performed on a MaXis impact, Bruker. Thin-layer chromatography was performed on silica gel plates (Sorbfil TLC, Krasnodar, Russia) to monitor the reactions. Spots were made visible with UV light. Column chromatography was performed with silica gel (Sigma-Aldrich, Burlington, MA, USA, high-purity grade, 60 Å/63–200 μm).

The spectrophotometric measurements were performed on a Cary-60 UV–Vis Spectrophotometer in 1 and 10 mm thick quartz cells. For photochemical measurements, the solutions were irradiated by an L8253 xenon lamp included in an LC-8 radiation unit (Hamamatsu, Shizuoka, Japan) at a medium radiation power through ultraviolet UFS-1 (from 240 nm to 400 nm with maximum transmission at 330 nm) and blue–green SZS-9 (from 380 nm to 580 nm with maximum transmission at 480 nm) filters.

The photoluminescence (PL) spectra of test compounds were registered using Horiba Jobin Yvon Fluorolog-3 spectrofluorometer (model FL-3-22) equipped with doublegrating monochromators, dual lamp housing containing a 450 W Xenon lamp and photomultiplier tube detector Hamamatsu R928 (Shizuoka, Japan). The PL spectra were corrected in all cases for source intensity (lamp and grating) and emission spectral response (detector and grating) by standard instrument correction provided in the instrument software FluorEssence 3.5 [41].

The detailed procedure of the synthesis and characterization of the products are given in the Supplementary Materials.

## 4. Conclusions

Thus, new photochromic spiropyrans containing in their structure a triphenylphosphonium cation with different spacer lengths were synthesized, and their photochromic and luminescent properties were studied. It is shown that all the synthesized compounds exhibit positive photochromism, as evidenced by their reversible photoinduced transformations under the influence of ultraviolet and visible radiation. The influence of structural factors on the spectral properties and kinetic characteristics of the photochromic transformations of the synthesized spirophotochromes was established. First, the positions of the absorption bands of the cyclic and merocyanine forms **12**–**16** slightly depend on the spacer length between the spiropyran and triphenylphosphonium fragments. Thus, the hypsochromic shift of the spectral bands in the series **12**–**16** is 4 nm for the spirocyclic form and 9 nm for the merocyanine form. Second, the measured relaxation rate constants of spiropyrans **12**–**16** from the open to the closed form differ several times. Thus, the results of the study demonstrate the possibility of controlling the efficiency of the photoinduced transformations of spiropyrans by varying the spacer length. Moreover, the presence of a positive photochromic effect with photoswitchable luminescence will make it possible to monitor the progress of various intracellular processes.

Summarizing the above, we can conclude that the synthesized cationic spiropyrans **12**–**16**, due to their high photosensitivity, displayed increased resistance to photodegradation and an ability to photoluminesce, determine the relevance of the development of research in the field of the synthesis of new structures and the need to continue further research into the study of their physicochemical properties and biological activity.

## Figures and Tables

**Table 1:** Spectral–kinetic characteristics of the studied photochromic compounds in spiropyran (SP) and merocyanine (MC) forms.

Compound	Form	λ_abs_ (nm) ^1^	k_1_ (s^−1^) ^2^	k_2_ (s^−1^) ^3^	τ_1/2_ (min) ^4^	S (rel.u.) ^5^
12	SP	225, 267, 335	7.5 × 10^−4^	0.1084	31.9	2.0
	MC	310, 364, 551				
13	SP	224, 267, 338	6.5 × 10^−4^	0.1166	94.2	2.1
	MC	311, 365, 547				
14	SP	225, 267, 339	6.8 × 10^−4^	0.264	99.1	2.0
	MC	311, 362, 542				
15	SP	225, 267, 338	2.8 × 10^−4^	0.246	65.0	2.2
	MC	309, 362, 543				
16	SP	225, 267, 339	4.0 × 10^−4^	0.2700	66.5	2.1
	MC	310, 361, 543				

^1^ The most characteristic absorption bands are given. ^2^ Rate constant for spontaneous dark decolorization of the merocyanine form of spiropyran at room temperature. ^3^ Rate constant for photobleaching of the merocyanine form of spiropyran at room temperature. ^4^ Efficiency of photodegradation (τ_1/2_), time of decrease by a factor of 2 of the value of optical density at the maximum of the absorption band of the photoinduced merocyanine form under continuous irradiation with UV light. ^5^ Photosensitivity of a photochromic compound (S), S = ∆D^phot^/D^max^, the ratio of the absorbance values at the maximum of the absorption band of the merocyanine form of spiropyran to the value of the optical density at the maximum of the absorption band of the spiropyran form.

## Data Availability

Data are contained within the article and Supplementary Materials.

## Supplementary Materials

The following supporting information can be downloaded at https://www.mdpi.com/article/10.3390/molecules29020368/s1, Figure S1.^1^H NMR spectra of compound **1** (400 MHz, CDCl_3_); Figure S2. ^13^C NMR spectra of compound **1** (100 MHz, CDCl_3_); Figure S3. ^1^H NMR spectra of compound **2** (400 MHz, CDCl_3_); Figure S4. ^13^C NMR spectra of compound **2** (100 MHz, CDCl_3_); Figure S5. ^1^H NMR spectra of compound **3** (400 MHz, CDCl_3_). Figure S6. ^13^C NMR spectra of compound **3** (100 MHz, CDCl_3_); Figure S7. ^1^H NMR spectra of compound **4** (400 MHz, CDCl_3_); Figure S8. ^13^C NMR spectra of compound **4** (100 MHz, CDCl_3_); Figure S9. ^1^H NMR spectra of compound **5** (400 MHz, CDCl_3_); Figure S10. ^13^C NMR spectra of compound **5** (100 MHz, CDCl_3_); Figure S11. ^1^H NMR spectra of compound **6** (400 MHz, CDCl_3_); Figure S12. ^1^H NMR spectra of compound **6** (100 MHz, CDCl_3_); Figure S13. ^1^H NMR spectra of compound **7** (400 MHz, CDCl_3_); Figure S14. ^13^C NMR spectra of compound **7** (100 MHz, CDCl_3_); Figure S15. ^1^H NMR spectra of compound **8** (400 MHz, CDCl_3_); Figure S16. ^13^C NMR spectra of compound **8** (100 MHz, CDCl_3_), Figure S17. ^1^H NMR spectra of compound **9** (400 MHz, CDCl_3_); Figure S18. ^13^C NMR spectra of compound **9** (100 MHz, CDCl_3_); Figure S19. ^1^H NMR spectra of compound **10** (400 MHz, CDCl_3_); Figure S20. ^13^C NMR spectra of compound **10** (100 MHz, CDCl_3_); Figure S21. ^1^H NMR spectra of compound **11** (400 MHz, CDCl_3_); Figure S22. ^13^C NMR spectra of compound **11** (100 MHz, CDCl_3_); Figure S23. ^1^H NMR spectra of compound **12** (500 MHz, CDCl_3_); Figure S24. ^13^C NMR spectra of compound **12** (125 MHz, CDCl_3_); Figure S25. HSQC spectra of compound **12** (500.17 MHz for ^1^H, 125.78 MHz for ^13^C, CDCl_3_); Figure S26. HMBC spectra of compound **12** (500.17 MHz for ^1^H, 125.78 MHz for ^13^C, CDCl_3_); Figure S27. ^31^P NMR spectra of compound **12** (202 MHz, CDCl_3_); Figure S28. ^1^H NMR spectra of compound **13** (500 MHz, CDCl_3_); Figure S29. ^13^C NMR spectra of compound **13** (125 MHz, CDCl_3_); Figure S30. ^31^P NMR spectra of compound **13** (202 MHz, CDCl_3_); Figure S31. ESI-HRMS spectra of compound **13**; Figure S32. Absorption spectra of **13** in ethanol in spiropyran (1) and merocyanine forms (2–14) measured before (1) and upon UV–irradiation (2) through a UFS-1 light filter and during bleaching in the dark (3–14). C = 10^–4^ M, l = 0.1 cm; Figure S33. ^1^H NMR spectra of compound **14** (500 MHz, CDCl_3_); Figure S34. ^13^C NMR spectra of compound **14** (125 MHz, CDCl_3_); Figure S35. ^31^P NMR spectra of compound **14** (202 MHz, CDCl_3_); Figure S36. ESI–HRMS spectra of compound **14**; Figure S37. Absorption spectra of **14** in ethanol in spiropyran (1) and merocyanine forms (2–17) measured before (1) and upon UV-irradiation (2) through a UFS-1 light filter and during bleaching in the dark (3–17). C = 10^–4^ M, l = 0.1 cm; Figure S38. ^1^H NMR spectra of compound **15** (500 MHz, CDCl_3_); Figure S39. ^13^C NMR spectra of compound **15** (125 MHz, CDCl_3_); Figure S40. ^31^P NMR spectra of compound **15** (202 MHz, CDCl_3_); Figure S41. ESI–HRMS spectra of compound **15**; Figure S42. Absorption spectra of **15** in ethanol in spiropyran (1) and merocyanine forms (2–14) measured before (1) and upon UV-irradiation (2) through a UFS-1 light filter and during bleaching in the dark (3–14). C = 10^–4^ M, l = 0.1 cm; Figure S43. ^1^H NMR spectra of compound **16** (500 MHz, CDCl_3_); Figure S44. ^13^C NMR spectra of compound **16** (125 MHz, CDCl_3_); Figure S45. ^31^P NMR spectra of compound **16** (202 MHz, CDCl_3_); Figure S46. ESI–HRMS spectra of compound **16**; Figure S47. Absorption spectra of **16** in ethanol in spiropyran (1) and merocyanine forms (2–17) measured before (1) and upon UV-irradiation (2) through a UFS-1 light filter and during bleaching in the dark (3–17). C = 10^–4^ M, l = 0.1 cm.
