# Supplementary material for: New Triphenylphosphonium Salts of Spiropyrans: Synthesis and Photochromic Properties
Source: Molecules. 2024 Jan 11;29(2):368. doi: 10.3390/molecules29020368 (PMC10821322; doi:10.3390/molecules29020368)
Supplement: Supplementary file 1 [file molecules-29-00368-s001.zip › molecules-2795169-supplementary.pdf]

Supplementary materials

New Triphenylphosphonium Salts of Spiropyrans: Synthesis and Photochromic Properties

A.A. Khuzin,[1]* D.I. Galimov,[1] L.L. Khuzina,[1] A.A. Tukhbatullin[1]

[1]*Institute of Petrochemistry and Catalysis Ufa Federal Research Center of the Russian Academy of Sciences, 141, Oktyabrya Prospect, 450075 Ufa, Russia*

* E-mail address: artur.khuzin@gmail.com.

Phenylhydrazine, 1,3-dibromopropane, 1,4-dibromobutane, 1,5-dibromopentane, 1,6-dibromohexane, 1,8-dibromooctane, 2-hydroxy-5-nitrobenzaldehyde, triethylamine, triphenylphosphine were purchased from Aldrich and used as received.

Synthesis of compound 1: A mixture of 60 g (1 eq) of phenylhydrazine and 60 ml (1 eq) of 3-methyl-2-butanone was refluxed in glacial acetic acid for 2.5 h. After cooling, the mixture was neutralized, the organic phase was separated and distilled. Yield: 93% of theoretical.

^{1}H NMR (400 MHz, $CDCl_3$): δ = 1.31 (s, 6H, $2CH_3$), 2.30 (s, 3H, $1CH_3$), 7.21 (t, J = 7.3 Hz,1H), 7.30 (m, 2H), 7.56 (d, J = 7.7 Hz, 1H) ppm. ^{13}C NMR (100 MHz, $CDCl_3$): δ = 15.22, 23.04, 53.57, 119.78, 121.31, 125.21, 127.62, 145.49, 153.21, 188.27 ppm.

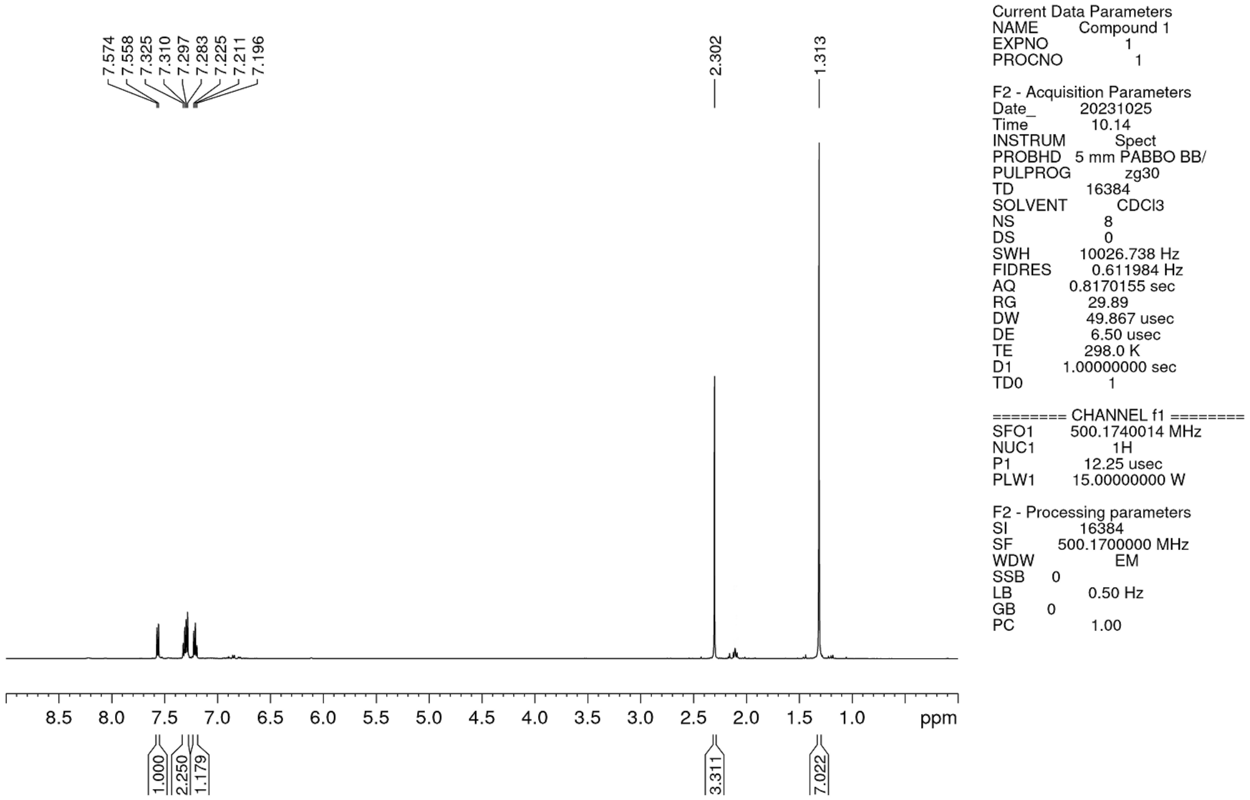

Figure S1. ^{1}H NMR spectra of compound **1** (400 MHz, $CDCl_3$).

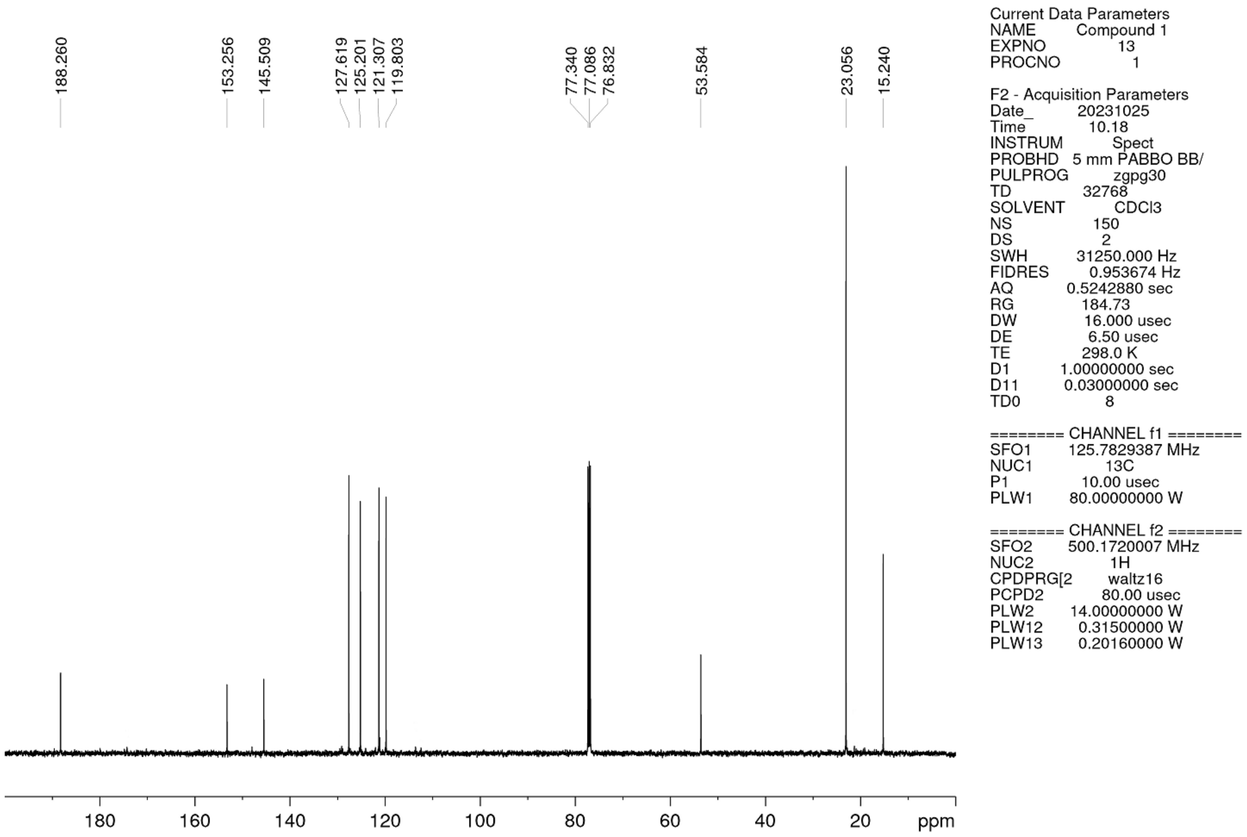

Figure S2. ^{13}C NMR spectra of compound **1** (100 MHz, $CDCl_3$).

General procedure for the synthesis of compounds 2-6: 2,3,3-Trimethyl-3H-indol **1** (1.0 eq) and dibromoalkane (3.0 eq) was refluxed in 95% acetonitrile for 8 h. Sequential purification via flash chromatography (acetone; dichloromethane:ethanole 10:1) afforded compounds **2-6** (30-35%) as a light yellow solid.

Compound 2: ^{1}H NMR (400 MHz, $CDCl_3$): δ = 1.32 (s, 6H), 1.88 (br. s, 2H), 2.01 (d, *J* = 3.0 Hz, 2H), 2.99 (br. s, 2H), 4.19 (br. s, 2H), 7.24 (m, 4H), 7.40 (d, *J* = 6.5 Hz, 1H) ppm. ^{13}C NMR (100 MHz, $CDCl_3$): δ = 16.40, 19.85, 23.25, 24.47, 46.02, 53.58, 114.57, 122.70, 128.94, 129.71, 141.19, 141.26, 195.23 ppm.

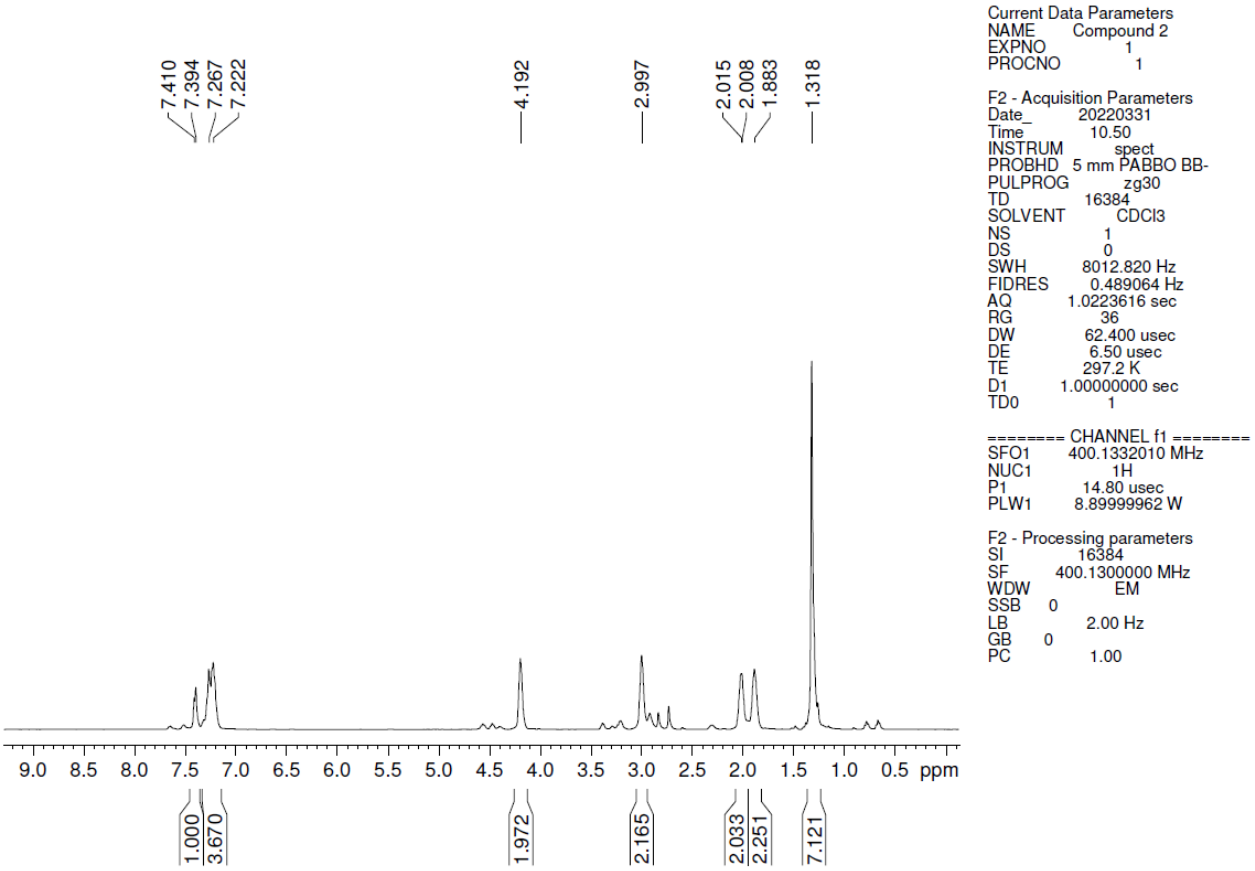

Figure S3. ^{1}H NMR spectra of compound **2** (400 MHz, $CDCl_3$).

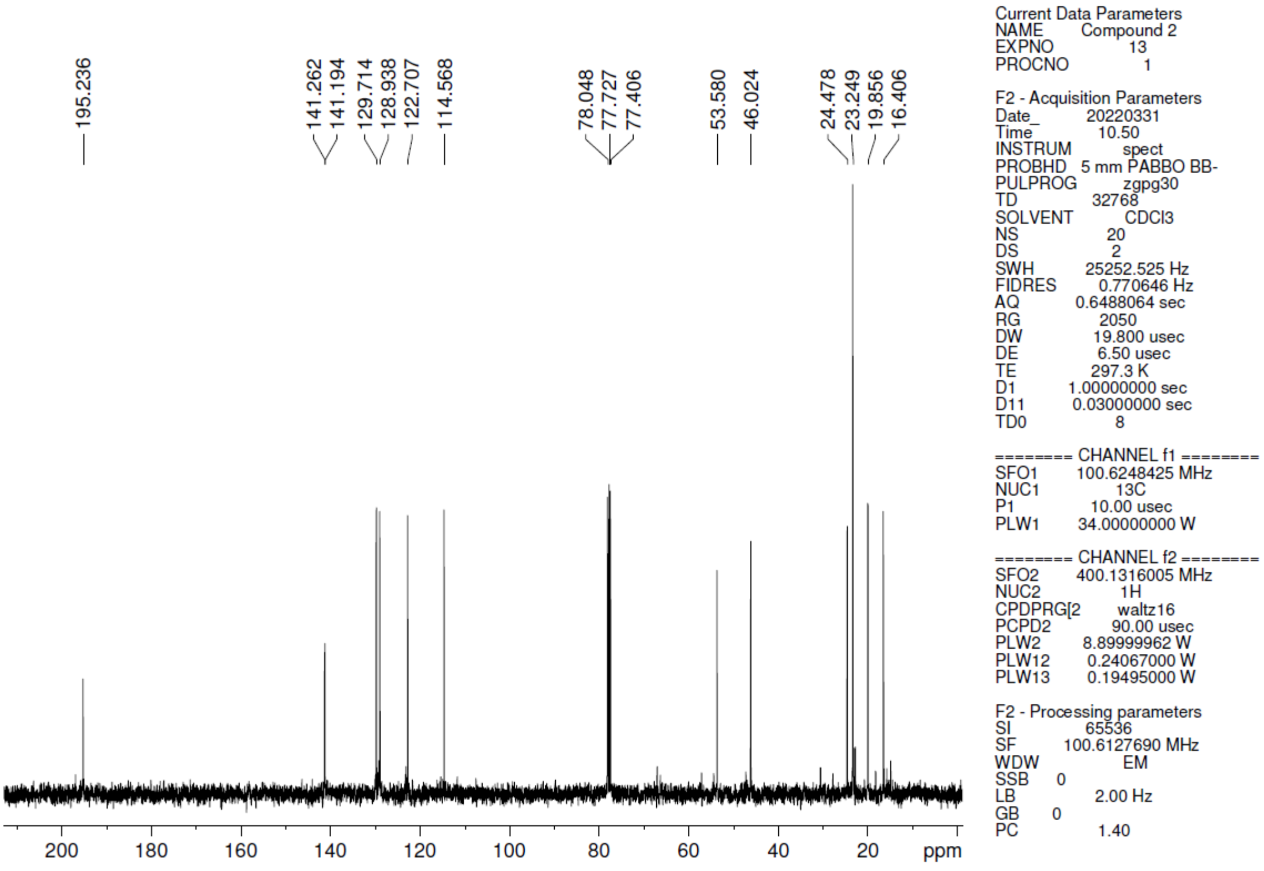

Figure S4. ^{13}C NMR spectra of compound **2** (100 MHz, $CDCl_3$).

Compound 3: ^{1}H NMR (400 MHz, $CDCl_3$): δ = 1.42 (s, 6H), 1.93 (br. s, 4H), 2.93 (s, 3H), 3.32 (t, *J* = 5.3 Hz, 2H), 4.59 (t, *J* = 6.6 Hz, 2H), 7.39 (m, 3H), 7.70 (m, 3H) ppm. ^{13}C NMR (100 MHz, $CDCl_3$): δ = 16.12, 22.92, 26.37, 29.12, 33.18, 48.19, 54.52, 115.52, 123.25, 129.43, 129.98, 140.71, 141.39, 196.18 ppm.

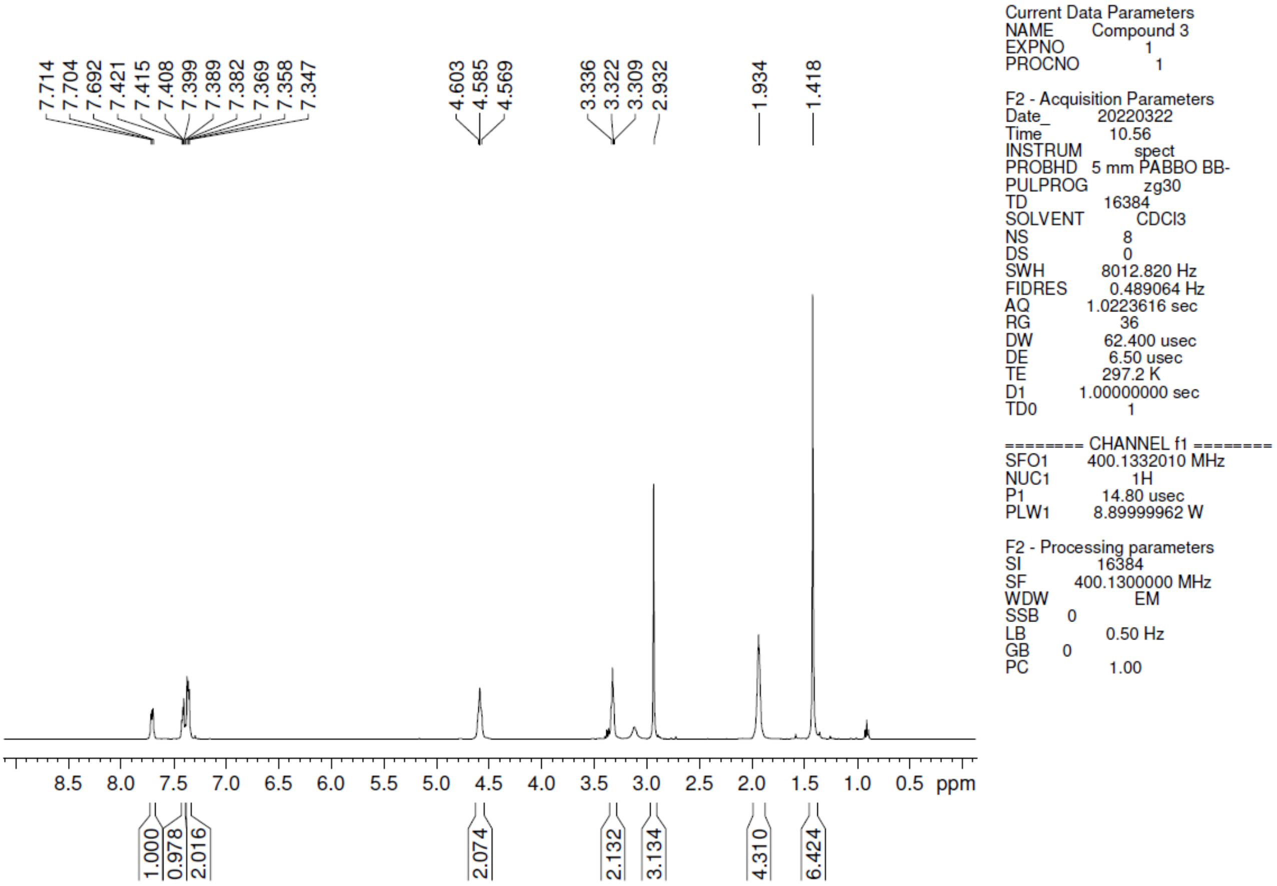

Figure S5. ^{1}H NMR spectra of compound **3** (400 MHz, $CDCl_3$).

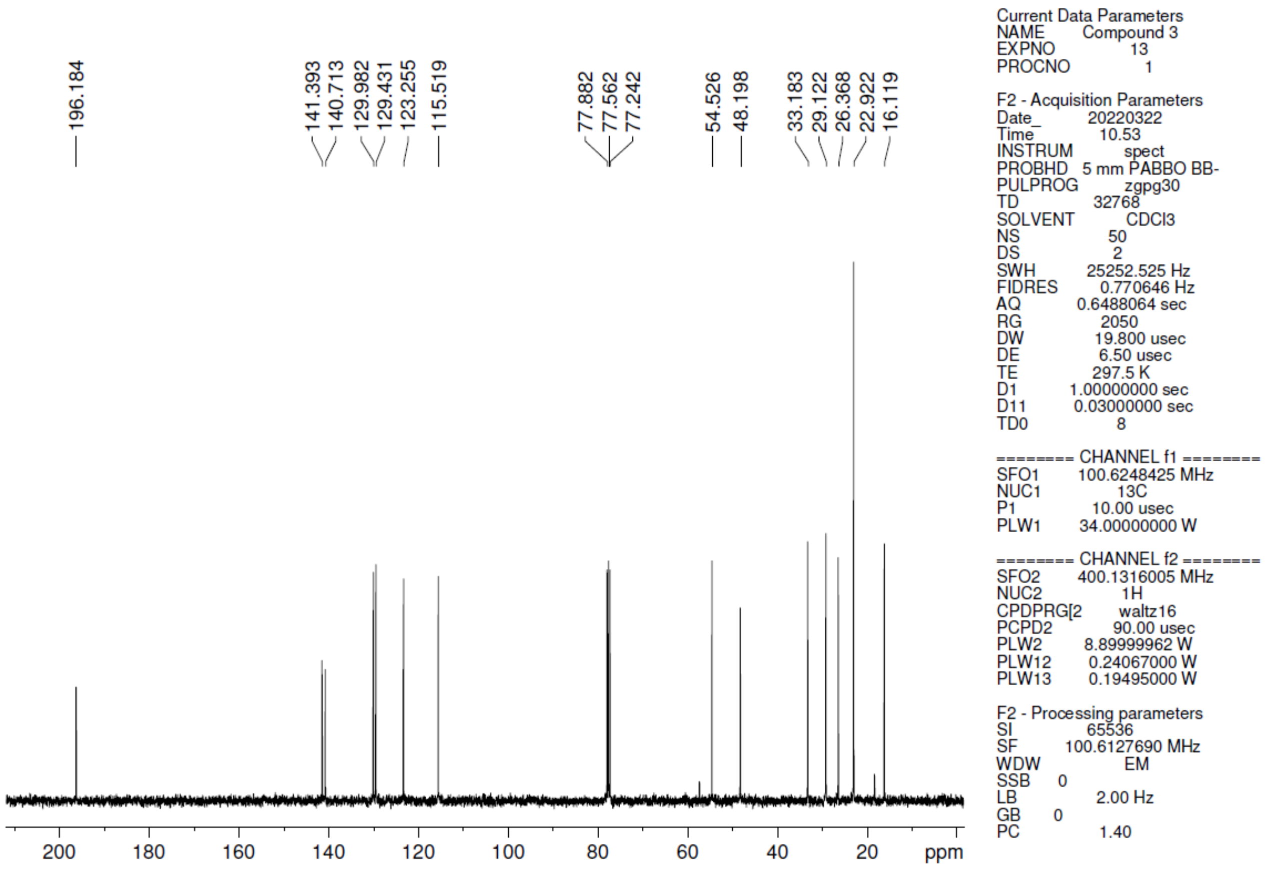

Figure S6. ^{13}C NMR spectra of compound **3** (100 MHz, $CDCl_3$).

Compound 4: ^{1}H NMR (400 MHz, $CDCl_3$): δ = 1.64 (s, 6H), 1.93 (dt, *J* = 15.6, 7.8 Hz, 2H), 2.02 (dt, *J* = 15.7, 7.9 Hz, 2H), 3.18 (s, 3H), 3.42 (t, *J* = 6.4 Hz, 2H) 4.81 (t, *J* = 7.6 Hz, 2H), 7.58 (s, 3H), 7.77 (d, *J* = 4.7 Hz, 1H) ppm. ^{13}C NMR (100 MHz, $CDCl_3$): δ = 16.52, 23.15, 25.14, 27.13, 31.64, 33.49, 49.39, 54.65, 115.50, 123.29, 129.57, 130.09, 140.97, 141.64, 196.31 ppm.

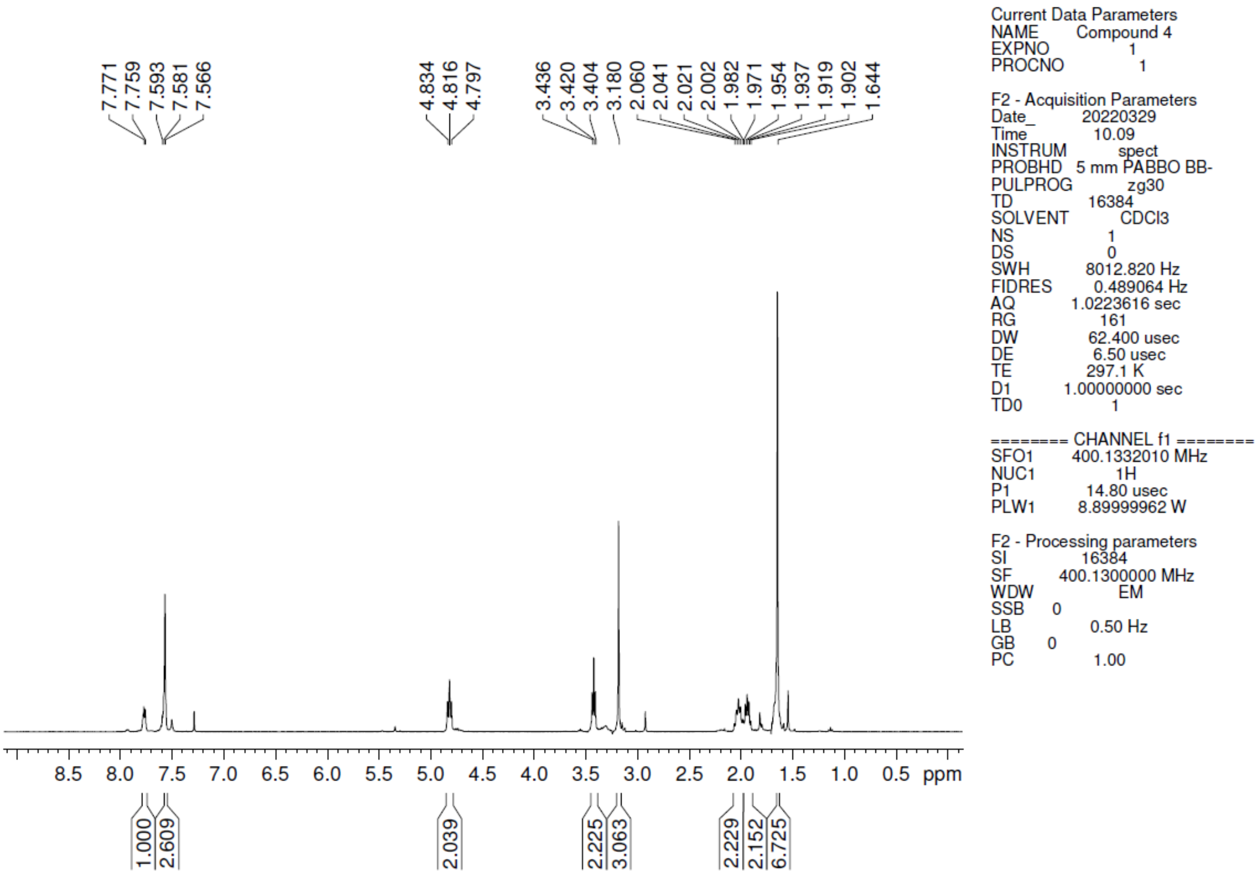

Figure S7. ^{1}H NMR spectra of compound **4** (400 MHz, $CDCl_3$).

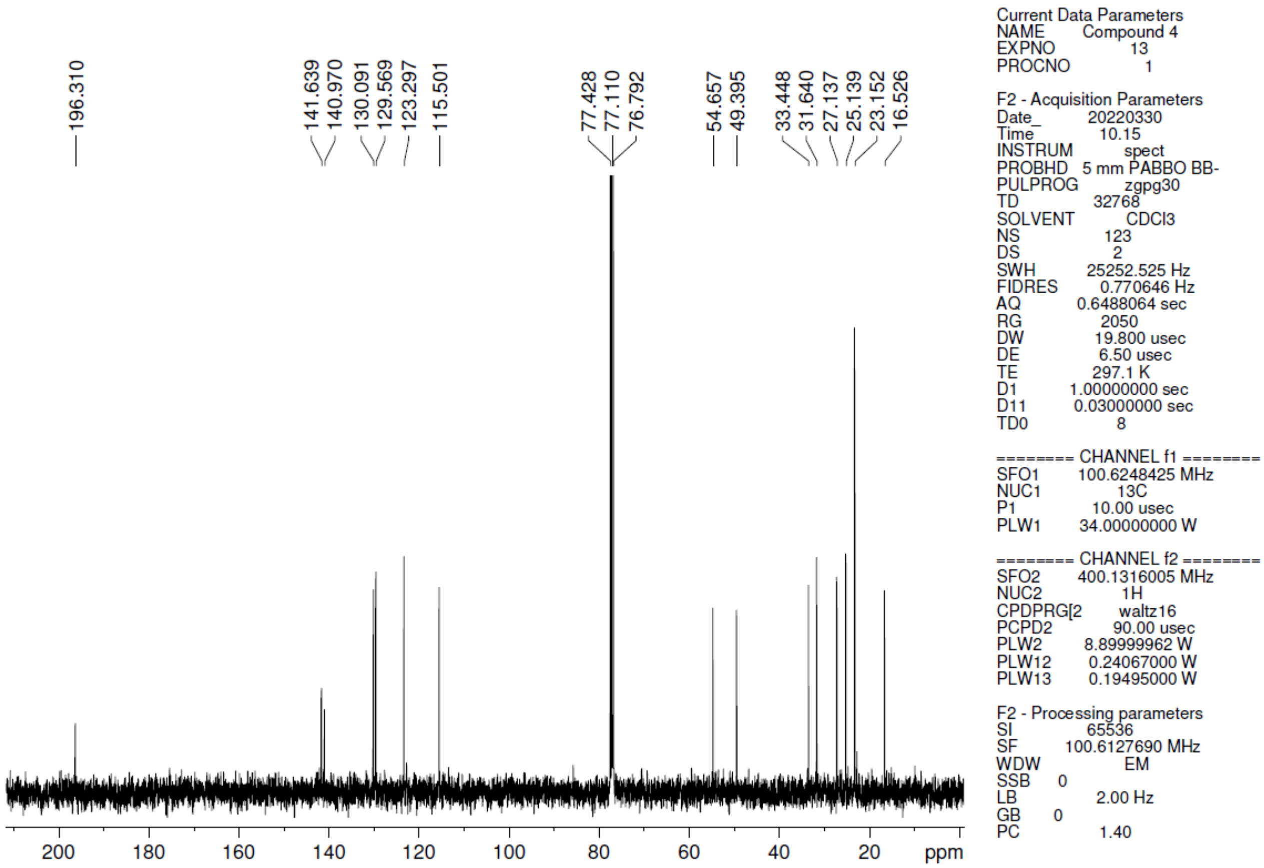

Figure S8. ^{13}C NMR spectra of compound **4** (100 MHz, $CDCl_3$).

Compound 5: ^{1}H NMR (400 MHz, $CDCl_3$): δ = 1.37 (m, 4H), 1.48 (s, 6 H), 1.70 (m, 2H), 1.82 (m, 2H), 2.96 (s, 3H), 3.24 (t, *J* = 6.6 Hz, 2H) 4.56 (t, *J* = 7.7 Hz, 2H), 7.42 (m, 2H), 7.46 (m, 1H), 7.62 (m, 1H) ppm. ^{13}C NMR (100 MHz, $CDCl_3$): δ = 16.13, 22.97, 25.74, 27.49, 27.84, 32.11, 33.83, 49.14, 54.55, 115.34, 123.34, 129.43, 130.02, 140.74, 141.51, 195.94 ppm.

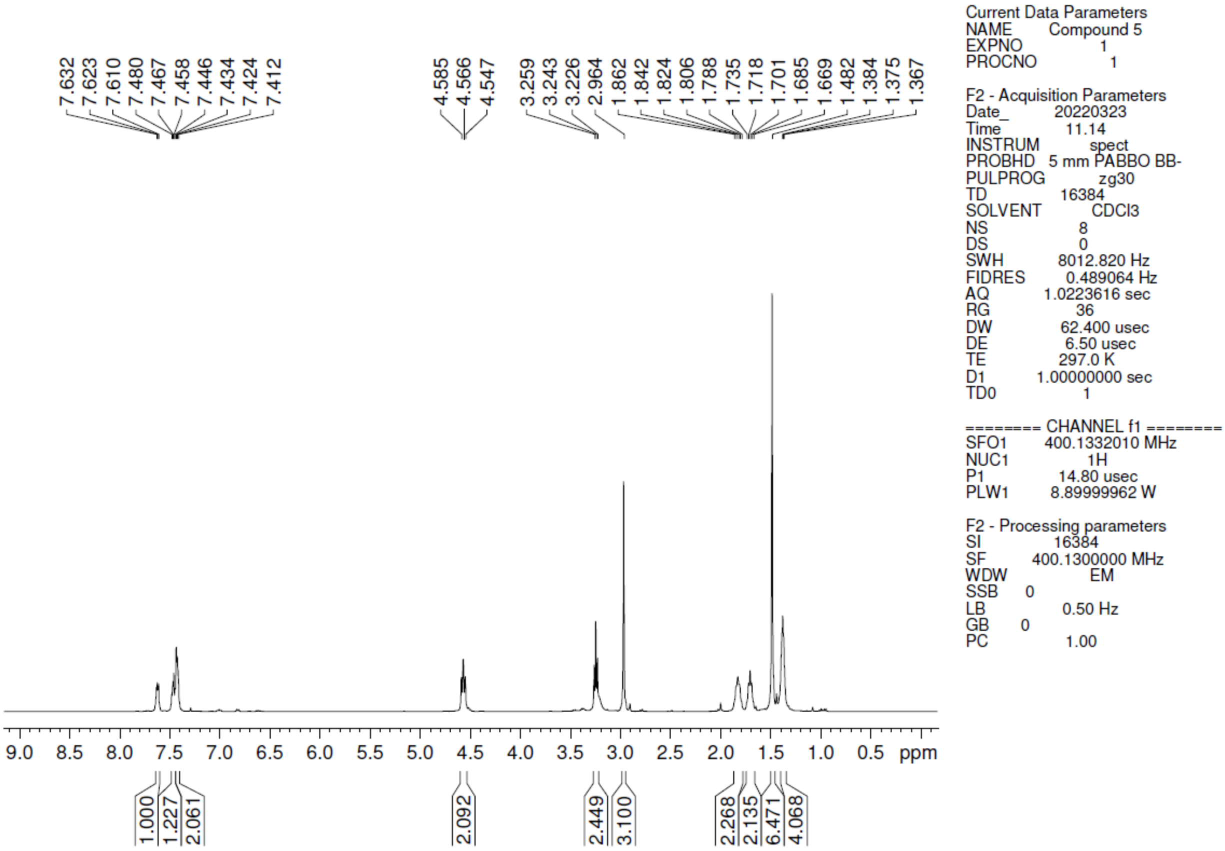

Figure S9. ^{1}H NMR spectra of compound **5** (400 MHz, $CDCl_3$).

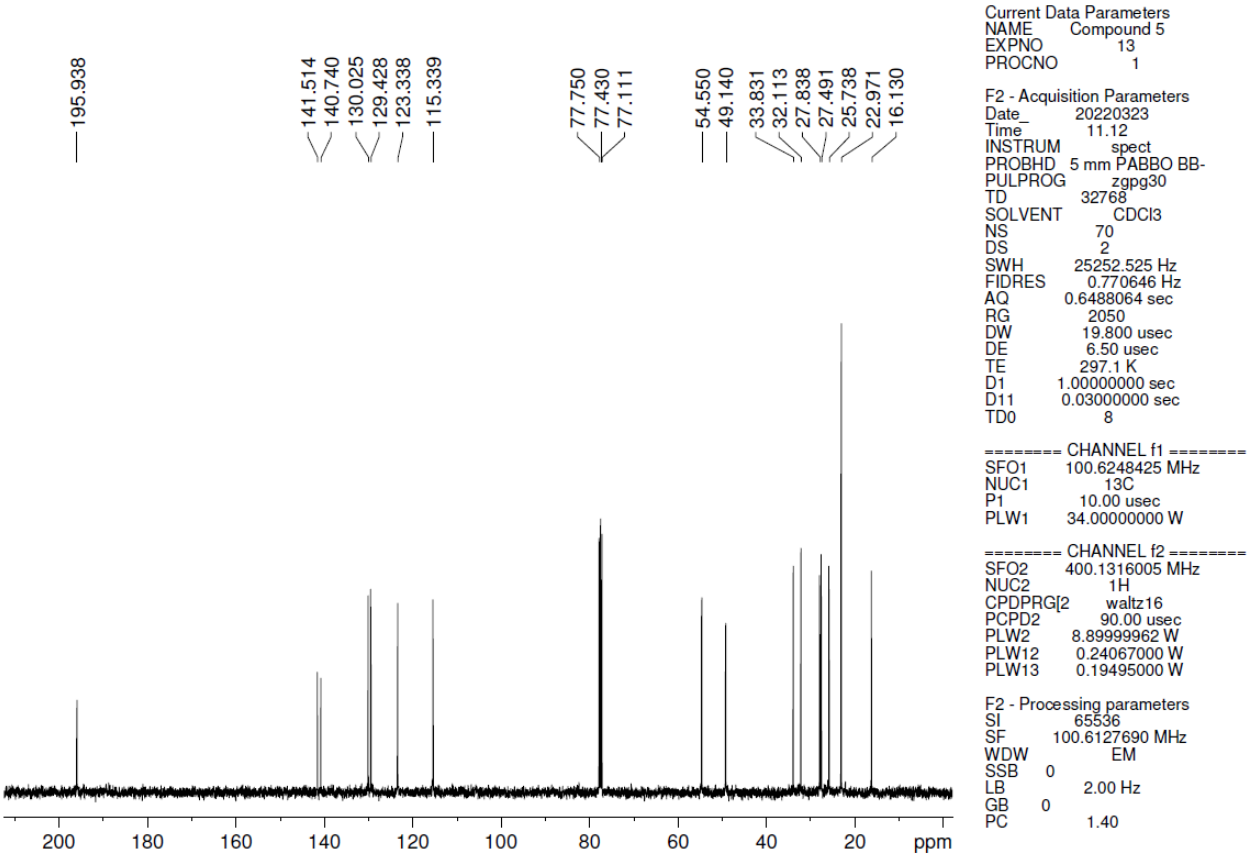

Figure S10. ^{13}C NMR spectra of compound **5** (100 MHz, $CDCl_3$).

Compound 6: ^{1}H NMR (400 MHz, $CDCl_3$): δ = 1.34 (m, 9H), 1.58 (s, 6 H), 1.76 (m, 2H), 1.89 (m, 2H), 2.96 (s, 3H), 3.08 (s, 3H), 3.34 (m, 3H), 4.67 (t, J = 7.4 Hz, 2H), 7.52 (m, 3H), 7.65 (m, 1H) ppm. ^{13}C NMR (100 MHz, $CDCl_3$): δ = 16.27, 23.05, 26.57, 27.79, 27.98, 28.30, 28.86, 32.49, 34.05, 49.47, 54.61, 115.30, 123.35, 129.46, 130.05, 140.89, 141.65, 195.95 ppm.

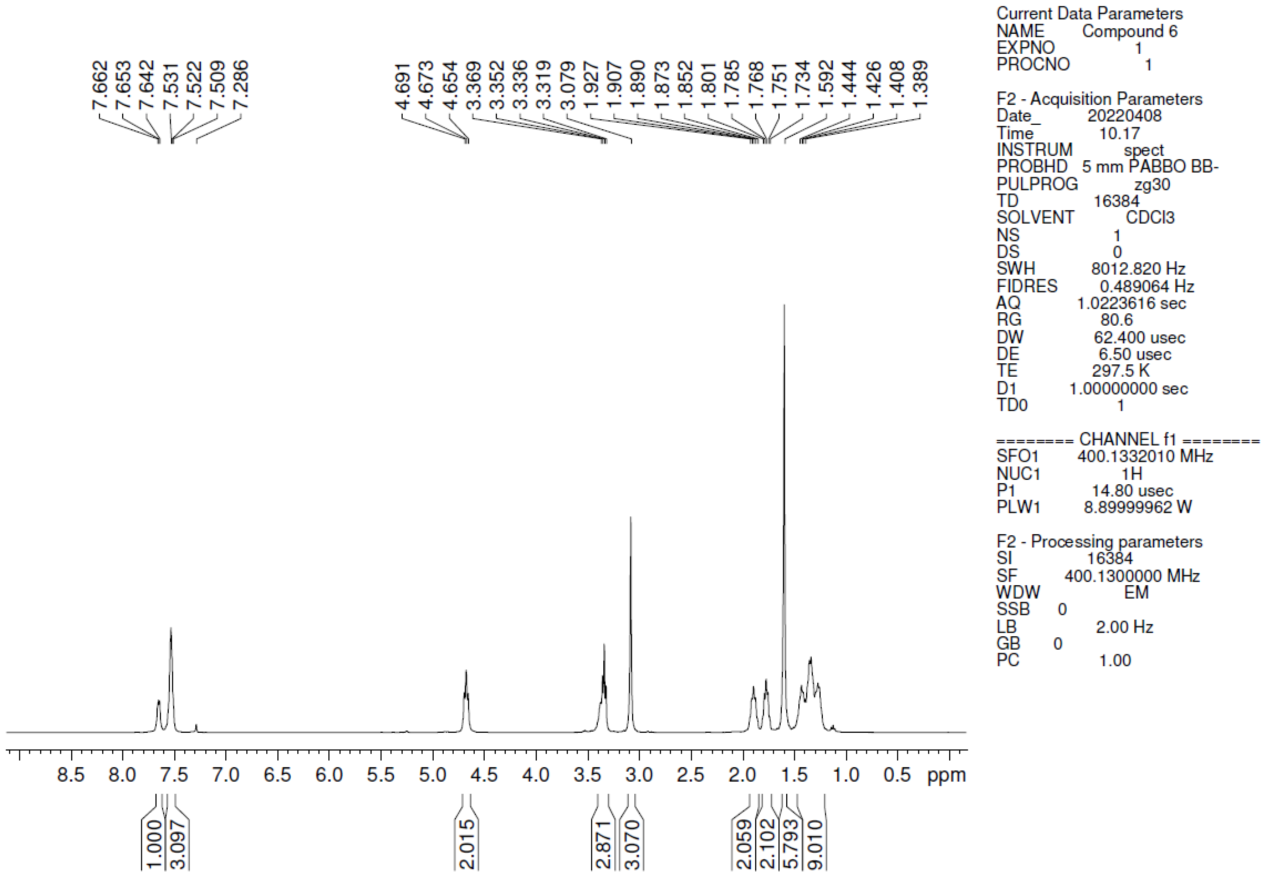

Figure S11. ^{1}H NMR spectra of compound **6** (400 MHz, $CDCl_3$).

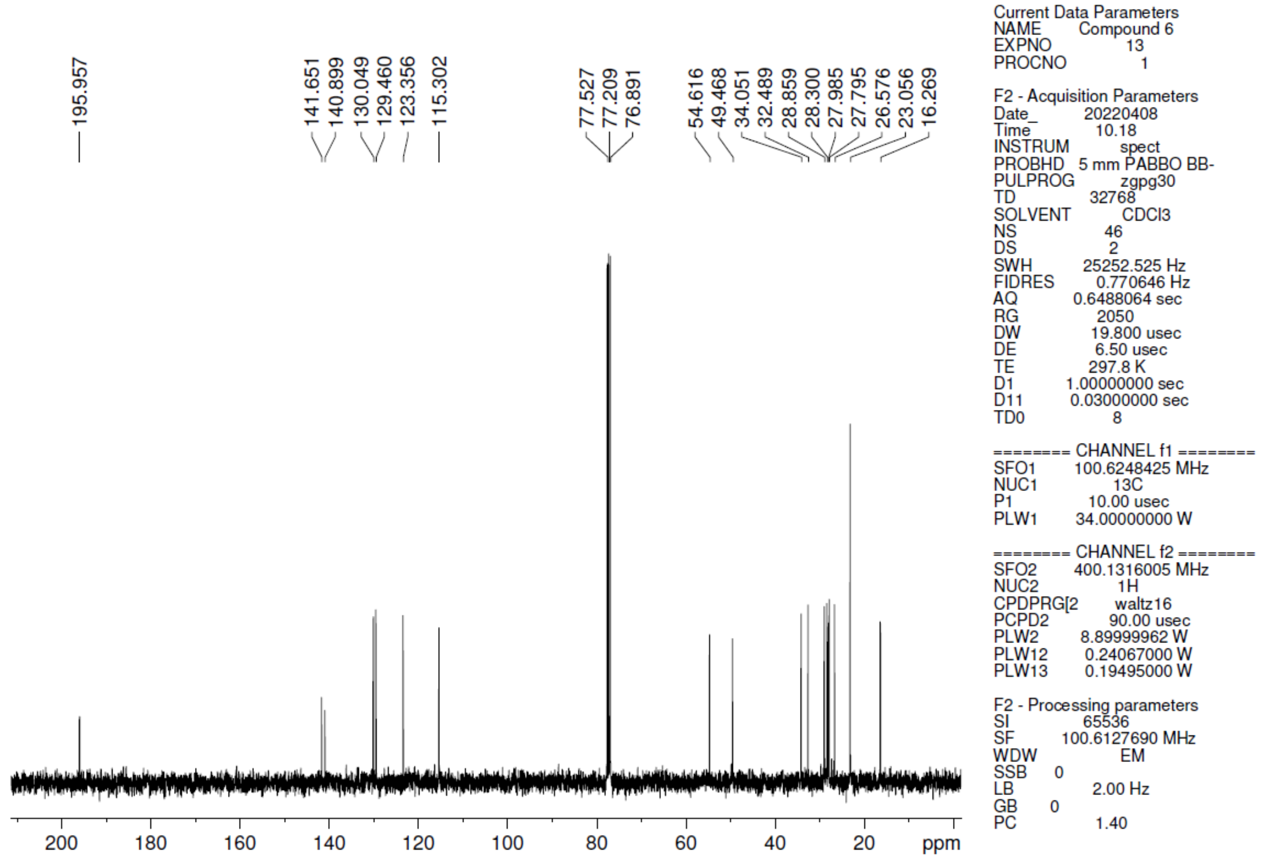

Figure S12. ^{1}H NMR spectra of compound **6** (100 MHz, $CDCl_3$).

General procedure for the synthesis of compounds 7-11: A solution of compounds **2-6** (1.0 eq), 2-hydroxy-5-nitrobenzaldehyde (1.0 eq) and Et_3N (1.1 eq) in EtOH were refluxed for 8 h. The white precipitate was removed. The crude product was purified by flash chromatography on silica gel with petroleum ether/EtOAc (10:1) as eluent to afford compouds **7-11** as light yellow solid (40-45%).

Compound 7: ^{1}H NMR (400 MHz, $CDCl_3$): δ = 1.22 (s, 3H), 1.32 (s, 3 H), 2.13 (m, 1H), 2.29 (m, 1H), 3.32 (m, 1H), 3.45 (m, 3H), 5.91 (d, *J* = 10.4 Hz, 1H), 6.68 (d, *J* = 7.8 Hz, 1H), 6.78 (d, *J* = 9.5 Hz, 1H), 6.93 (t, *J* = 7.4 Hz, 1H), 6.97 (d, *J* = 10.4 Hz, 1H), 7.13 (d, *J* = 7.1 Hz, 1H), 7.23 (t, *J* = 7.6 Hz, 1H), 8.04 (s, 1H), 8.06 (d, *J* = 2.5 Hz, 1H) ppm. ^{13}C NMR (100 MHz, $CDCl_3$): δ = 19.90, 25.97, 31.03, 31.77, 42.01, 52.65, 106.68, 106.86, 115.56, 118.52, 119.84, 121.74, 121.87, 122.80, 125.96, 127.84, 128.49, 136.03, 141.07, 146.83, 159.43 ppm.

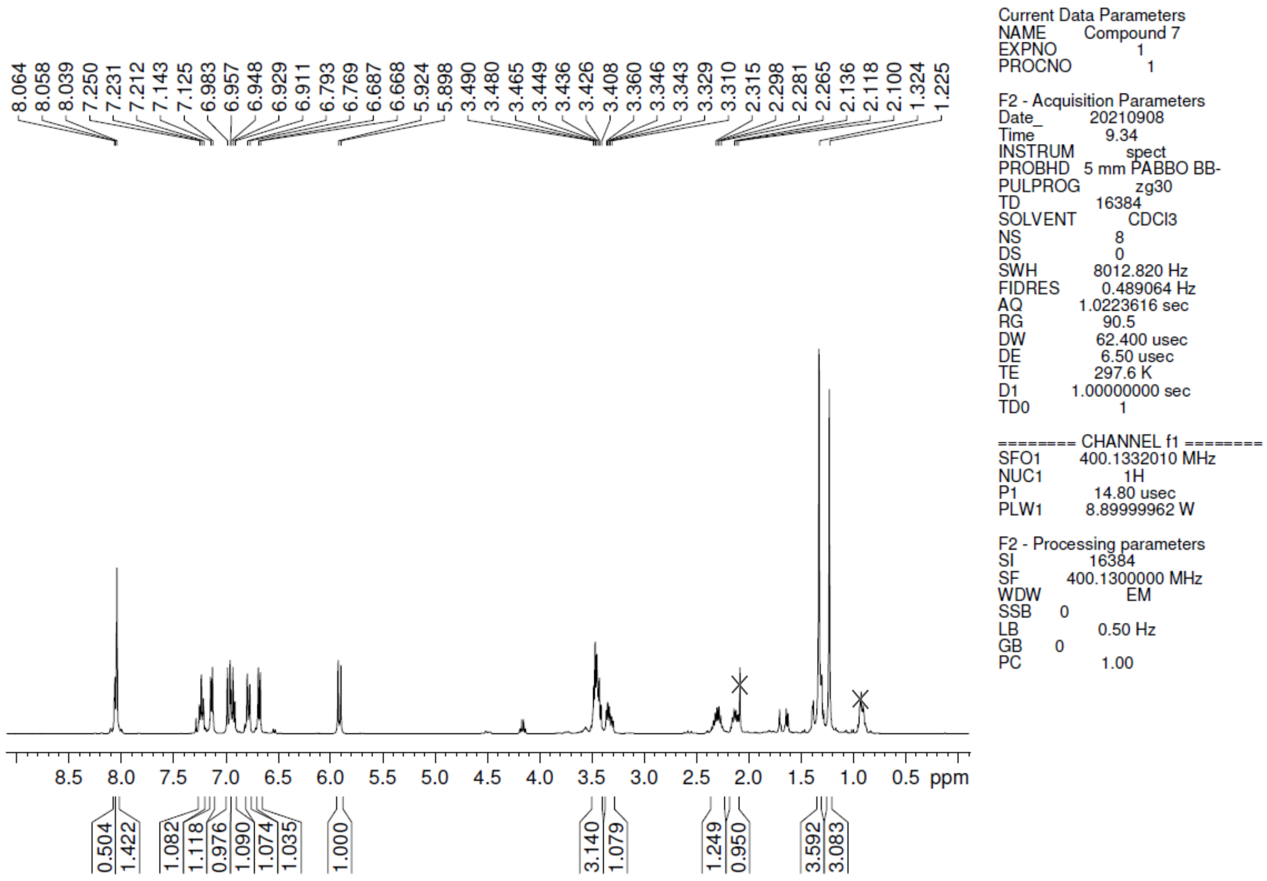

Figure S13. ^{1}H NMR spectra of compound **7** (400 MHz, $CDCl_3$).

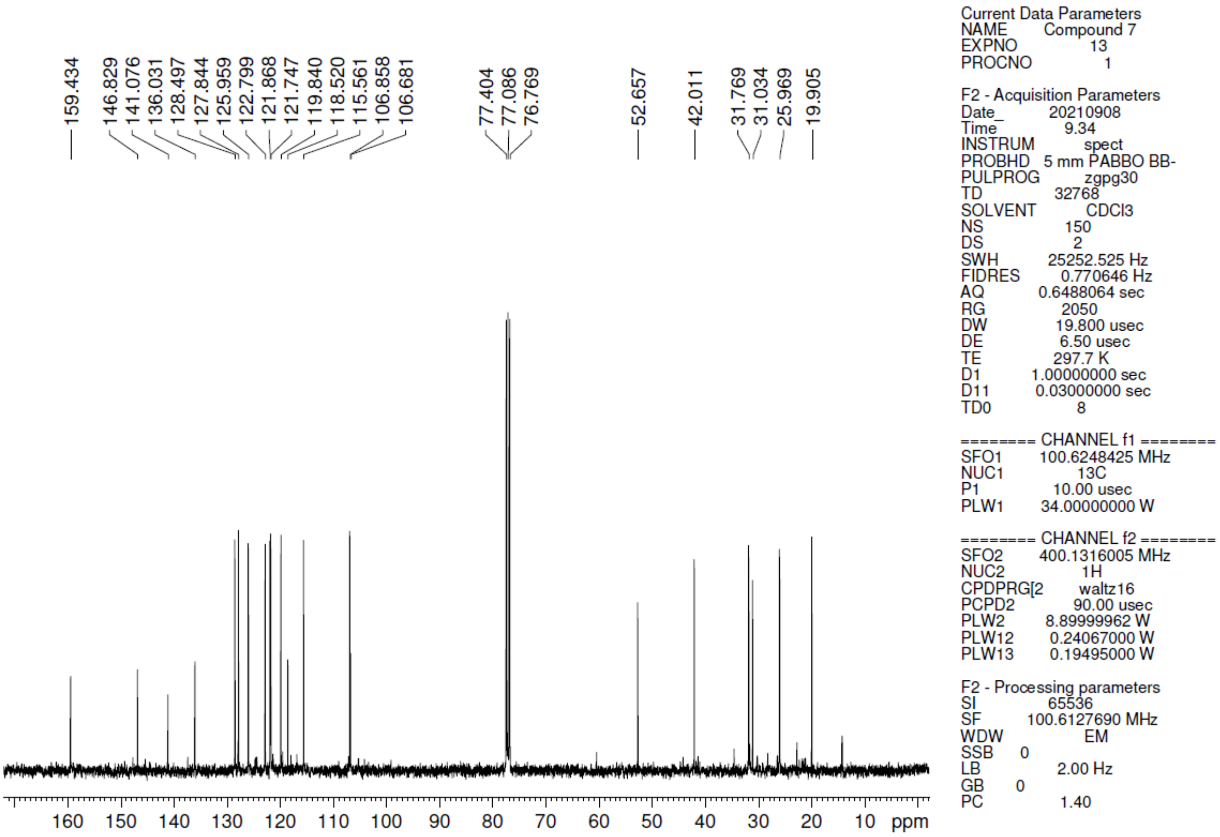

Figure S14. ^{13}C NMR spectra of compound **7** (100 MHz, $CDCl_3$).

Compound 8: ^{1}H NMR (400 MHz, $CDCl_3$): δ = 1.22 (s, 3H), 1.32 (s, 3 H), 1.88 (m, 4H), 3.22 (m, 2H), 3.41 (m, 2H), 5.90 (d, *J* = 10.4 Hz, 1H), 6.61 (d, *J* = 7.8 Hz, 1H), 6.77 (d, *J* = 9.3 Hz, 1H), 6.91 (t, *J* = 7.4 Hz, 1H), 6.95 (d, *J* = 10.4 Hz, 1H), 7.12 (d, *J* = 7.1 Hz, 1H), 7.22 (t, *J* = 7.6 Hz, 1H), 8.03 (s, 1H), 8.05 (d, *J* = 2.5 Hz, 1H) ppm. ^{13}C NMR (100 MHz, $CDCl_3$): δ = 19.90, 26.02, 27.68, 30.47, 33.33, 42.99, 52.65, 106.70, 115.57, 118.45, 119.61, 121.79, 121.86, 122.77, 125.95, 127.82, 128.29, 135.97, 140.99, 146.97, 159.57 ppm.

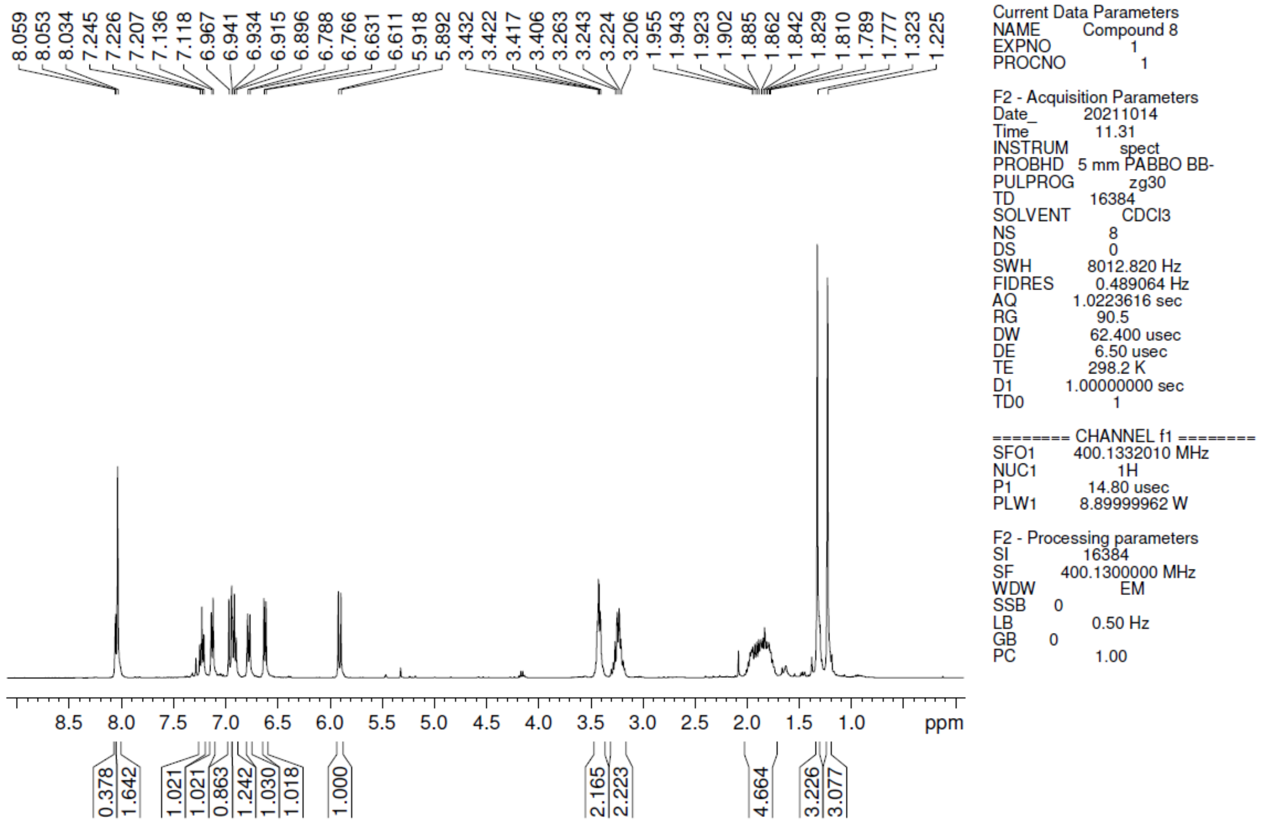

Figure S15. ^{1}H NMR spectra of compound **8** (400 MHz, $CDCl_3$).

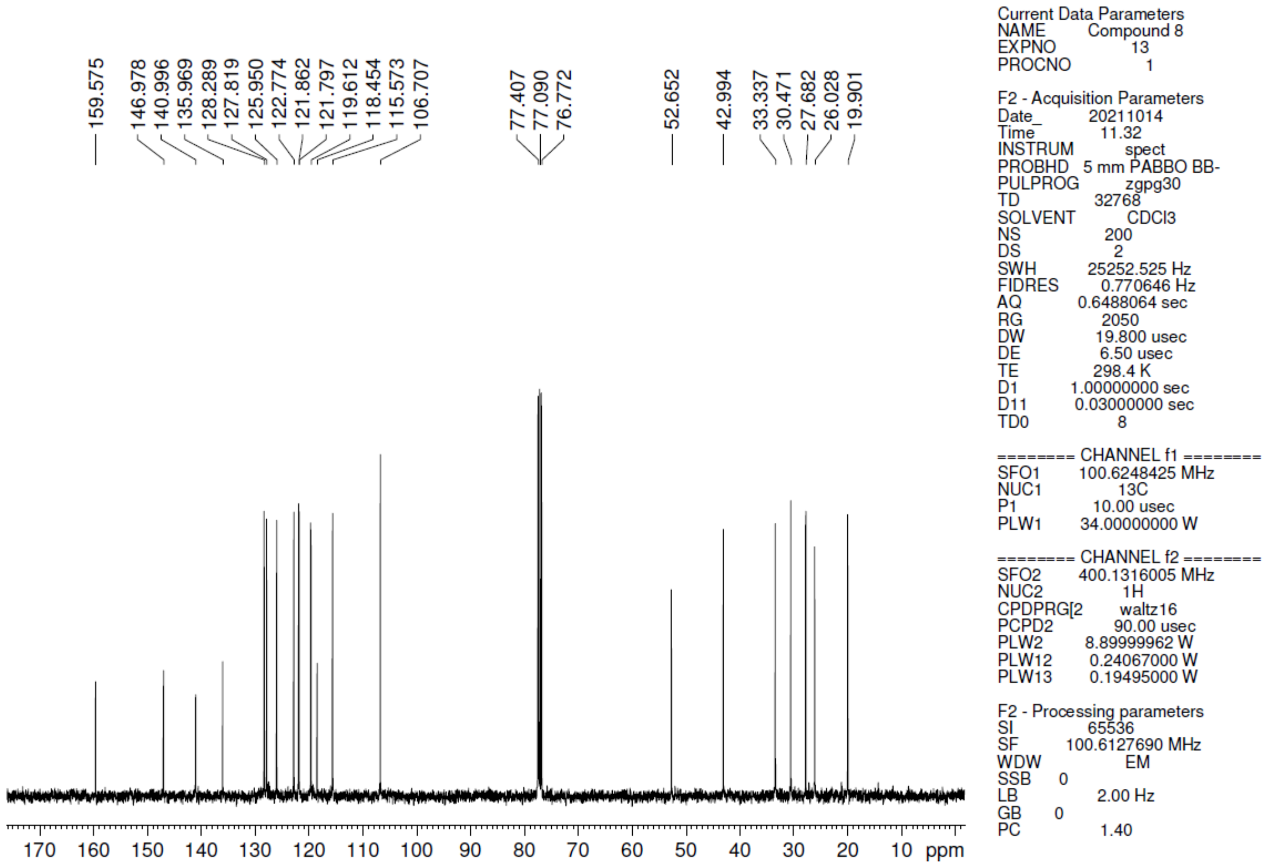

Figure S16. ^{13}C NMR spectra of compound **8** (100 MHz, $CDCl_3$).

Compound 9: ^{1}H NMR (400 MHz, $CDCl_3$): δ = 1.22 (s, 3H), 1.31 (s, 3 H), 1.51 (m, 2H), 1.59 (m, 1H), 1.72 (m, 1H), 1.88 (m, 2H), 3.20 (m, 2H), 3.41 (t, *J* = 6.5 Hz, 2H), 5.91 (d, *J* = 10.3 Hz, 1H), 6.60 (d, *J* = 7.7 Hz, 1H), 6.77 (d, *J* = 9.2 Hz, 1H), 6.90 (t, *J* = 7.4 Hz, 1H), 6.95 (d, *J* = 10.4 Hz, 1H), 7.12 (d, *J* = 7.1 Hz, 1H), 7.22 (t, *J* = 7.5 Hz, 1H), 8.04 (d, *J* = 7.3 Hz, 2H) ppm. ^{13}C NMR (100 MHz, $CDCl_3$): δ = 19.88, 25.87, 26.03, 28.12, 32.41, 33.70, 43.55, 52.65, 106.66, 106.75, 115.54, 118.48, 119.45, 121.76, 121.99, 122.76, 125.92, 127.77, 128.21, 135.97, 140.92, 147.04, 159.64 ppm.

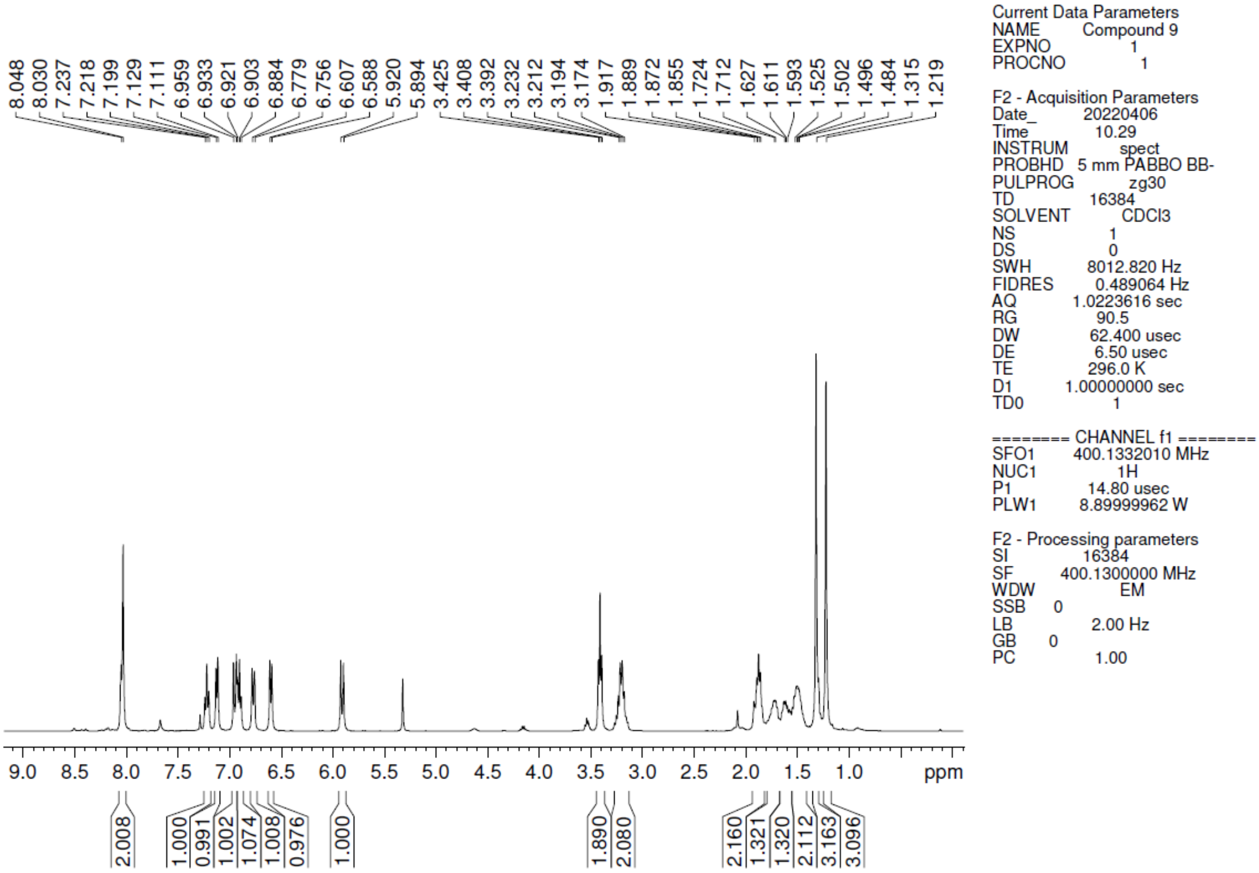

Figure S17. ^{1}H NMR spectra of compound **9** (400 MHz, $CDCl_3$).

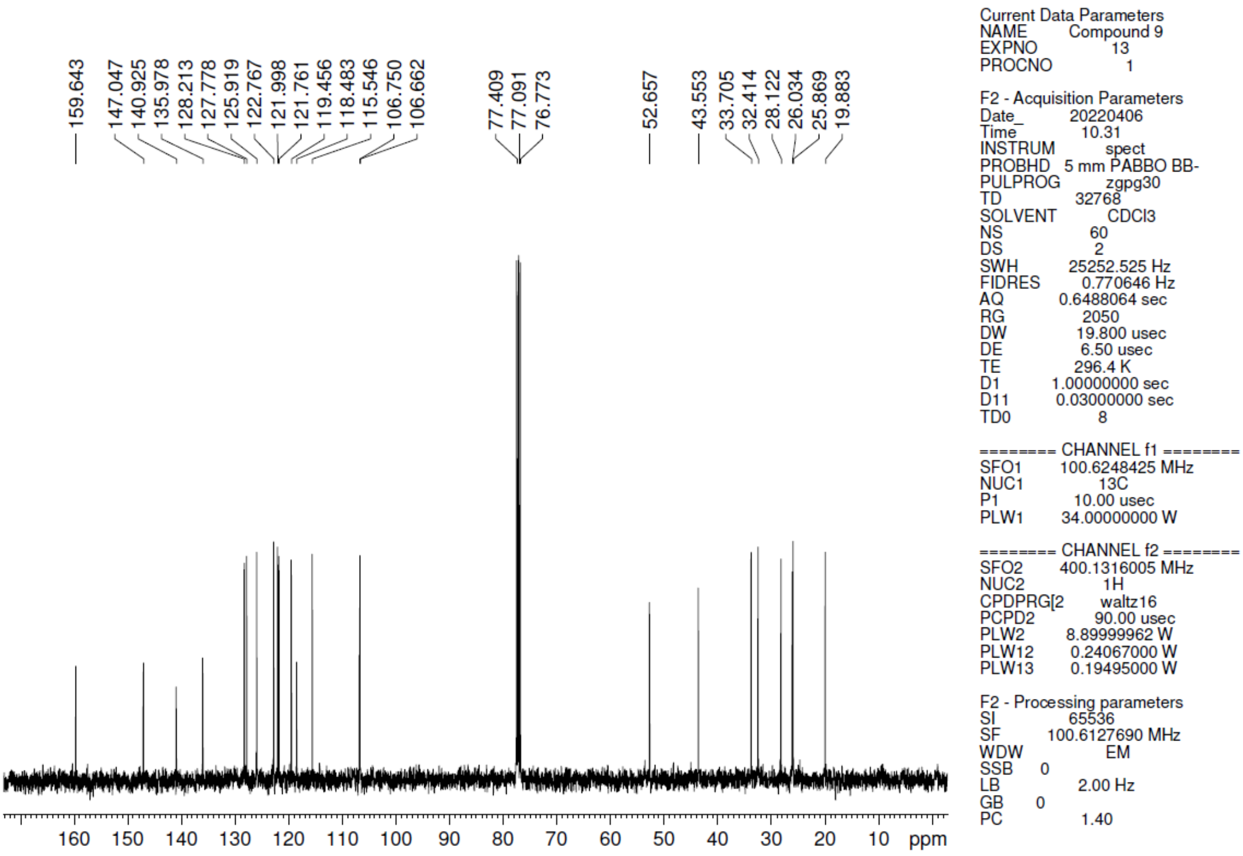

Figure S18. ^{13}C NMR spectra of compound **9** (100 MHz, $CDCl_3$).

Compound 10: ^{1}H NMR (400 MHz, $CDCl_3$): δ = 1.23 (s, 3H), 1.30 (s, 3 H), 1.37 (m, 2H), 1.45 (m, 3H), 1.58 (m, 2H), 1.71 (m, 2H), 1.85 (m, 2H), 3.19 (m, 2H), 3.40 (t, *J* = 6.7 Hz, 2H), 5.89 (d, *J* = 10.4 Hz, 1H), 6.59 (d, *J* = 7.7 Hz, 1H), 6.76 (d, *J* = 8.6 Hz, 1H), 6.89 (t, *J* = 7.4 Hz, 1H), 6.94 (d, *J* = 10.4 Hz, 1H), 7.11 (d, *J* = 7.2 Hz, 1H), 7.21 (t, *J* = 7.5 Hz, 1H), 8.03 (s, 1H), 8.05 (d, *J* = 2.5 Hz, 1H) ppm. ^{13}C NMR (100 MHz, $CDCl_3$): δ = 19.86, 26.05, 26.47, 27.93, 28.80, 32.67, 33.81, 43.60, 52.64, 106.65, 106.74, 115.55, 118.49, 119.37, 121.72, 121.99, 122.74, 125.90, 127.76, 128.16, 135.95, 140.90, 147.09, 159.67 ppm.

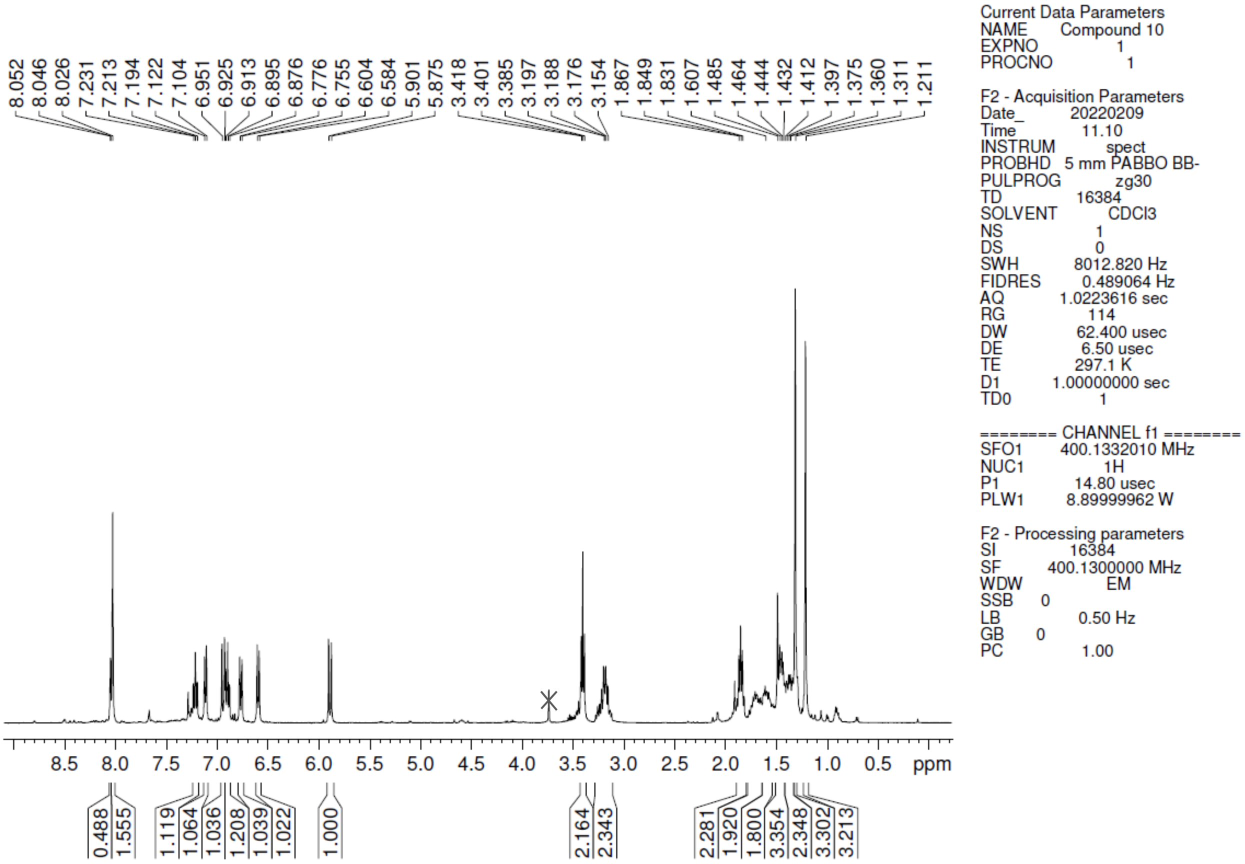

Figure S19. ^{1}H NMR spectra of compound **10** (400 MHz, $CDCl_3$).

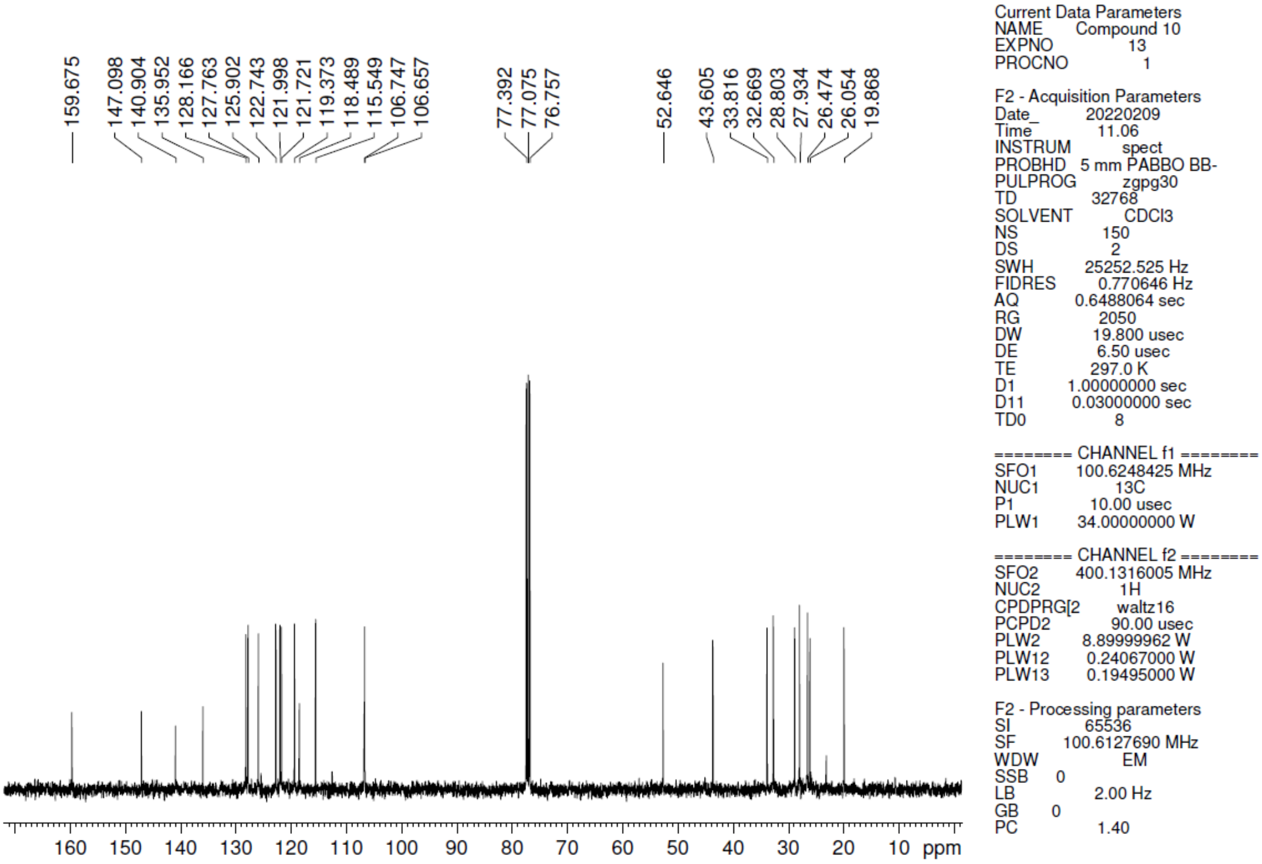

Figure S20. ^{13}C NMR spectra of compound **10** (100 MHz, $CDCl_3$).

Compound 11: ^{1}H NMR (400 MHz, $CDCl_3$): δ = 1.20 (s, 3H), 1.30 (s, 9H), 1.42 (br. s, 2H), 1.56 (br. s, 1H), 1.67 (br. s, 2H), 1.85 (m, 2H), 1.16 (m, 2H), 3.41 (t, *J* = 6.6 Hz, 2H), 5.88 (d, *J* = 10.3 Hz, 1H), 6.59 (d, *J* = 7.7 Hz, 1H), 6.76 (d, *J* = 8.5 Hz, 1H), 6.90 (dd, *J* = 19.1, 9.0 Hz, 2H), 7.10 (d, *J* = 7.0 Hz, 1H), 7.21 (t, *J* = 7.5 Hz, 1H), 8.02 (s, 1H), 8.04 (s, 1H) ppm. ^{13}C NMR (100 MHz, $CDCl_3$): δ = 19.85, 26.04, 27.19, 28.04, 28.65, 28.91, 29.19, 32.70, 33.97, 43.73, 52.64, 106.65, 106.76, 115.54, 118.50, 119.28, 121.68, 122.07, 122.70, 125.86, 127.74, 128.07, 135.93, 140.87, 147.13, 159.71 ppm.

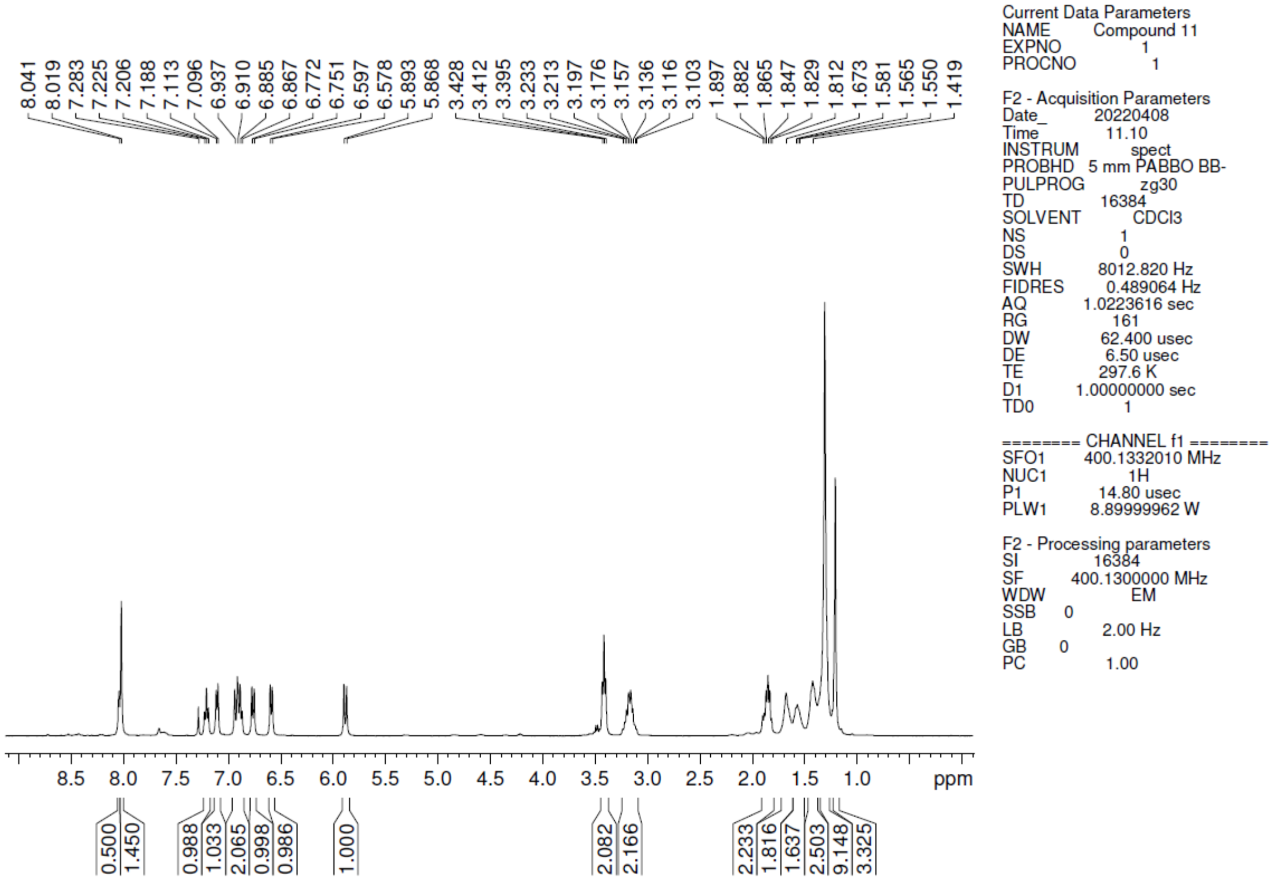

Figure S21. ^{1}H NMR spectra of compound **11** (400 MHz, $CDCl_3$).

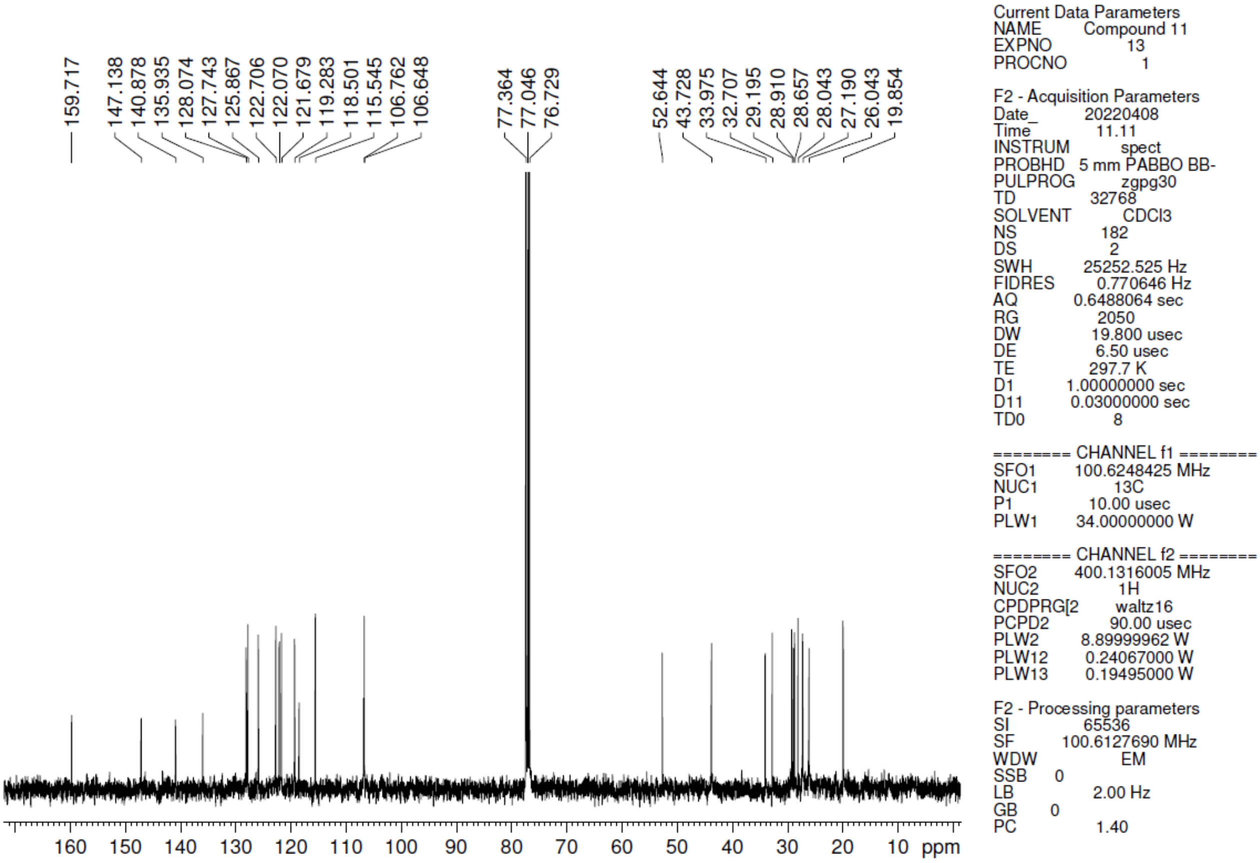

Figure S22. ^{13}C NMR spectra of compound **11** (100 MHz, $CDCl_3$).

General procedure for the synthesis of compounds 12-16: A solution of compounds **7-11** (1.0 eq) and triphenylphosphine (5.0 eq) in acetonitrile were refluxed for 16 h. After cooling, precipitated triphenylphosphine was removed by filtration. The remaining reaction mass after evaporation under reduced pressure was subjected to sequential purification by flash chromatography (petroleum ether/EtOAc (10:1); dichloromethane:ethanol (20:1)) to obtain the corresponding compounds **12-16** (68-76%) in the form of a dark cherry-colored solid.

The NMR spectra presented in this paper contain the proper number of signals corresponding to the structure of **12**. Thus, in the NMR spectrum on 1H nuclei of compound **12** in $CDCl_3$, two singlet signals at 1.09 and 1.22 ppm. belong to the protons of the methyl groups at the carbon atom of the indole ring. Multiplet signals in the region of 1.73–2.06, 3.61–3.88 and 3.79–4.19 ppm. belong to the signals of the protons of the carbon atoms of the alkyl chain of the spacer. A characteristic signal for the presence of a spirocyclic structure is a doublet in the olefinic region with a chemical shift of 5.83 ppm. with a spin–spin interaction constant of 10.4 Hz, belonging to the CH–group at the spiro atom in the pyran part of the molecule. In the low-field region in the intervals 7.60–7.64 and 7.74–7.79 ppm. signals from the hydrogen atoms of the aromatic rings of the triphenylphosphonium fragment of the molecule are observed (see Supplementary info).

In the ^{13}C NMR spectrum of compound **12** in $CDCl_3$, a characteristic signal of the spirocyclic carbon atom is observed at 107.35 ppm, which correlates in the HMBC ^{1}H–^{13}C spectrum with the signal of the gem–methyl group protons, as well as the C3' and C4' protons. The downfield signal at 159.09 belongs to the C9' carbon atom, covalently bonded to the oxygen atom of the pyran fragment of the molecule. In addition, due to the presence of a phosphorus atom, a doubling of the signals of the alkyl fragment of the molecule is observed in the ^{13}C NMR spectrum.

Compound 12: ^{1}H NMR (500 MHz, $CDCl_3$): δ = 1.09 (s, 3H), 1.22 (s, 3H), 1.73 (m, 2H), 1.87 (m, 2H), 2.06 (m, 1H), 3.61 (m, 1H), 3.79 (m, 1H), 3.88 (m, 1H), 4.19 (m, 1H), 5.83 (d, J = 10.4 Hz, 1H), 6.49 (d, J = 8.9 Hz, 1H), 6.74 (d, J = 7.8 Hz, 1H), 6.84 (d, J = 7.4 Hz, 1H), 6.94 (d, J = 10.4 Hz, 1H), 7.04 (d, J = 7.2 Hz, 1H), 7.14 (t, J = 7.6 Hz, 1H), 7.63 (m, 6H), 7.77 (m, 9H), 7.87 (dd, J_1 = 8.9, J_2 = 2.7 Hz, 1H), 7.92 (d, J = 2.7 Hz, 1H) ppm. ^{13}C NMR (125 MHz, $CDCl_3$): δ = 19.69, 20.15, 20.57, 21.44, 26.03, 29.68, 43.12, 43.28, 52.89, 106.68, 107.35, 115.02, 117.84, 118.52, 118.61, 119.63, 121.71, 121.85, 122.92, 125.55, 128.09, 128.78, 130.37, 130.47, 133.55, 133.63, 134.97, 134.99, 135.72, 140.82, 146.03, 159.09 ppm.

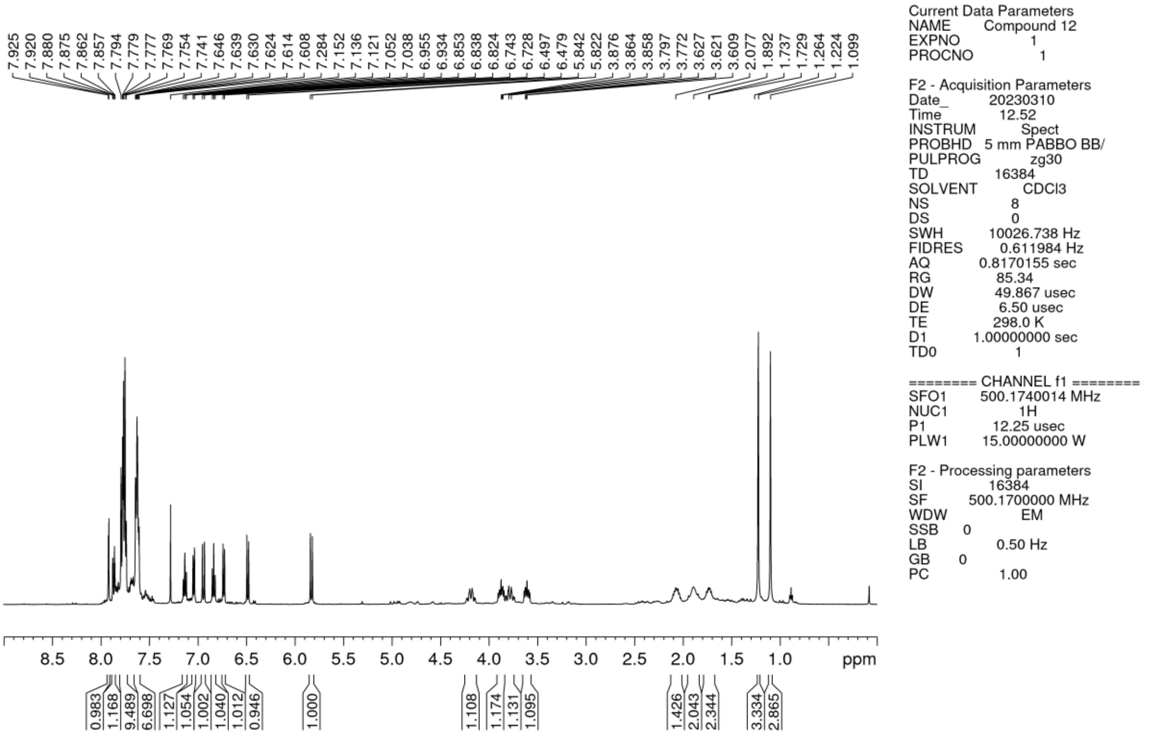

Figure S23. ^{1}H NMR spectra of compound **12** (500 MHz, $CDCl_3$).

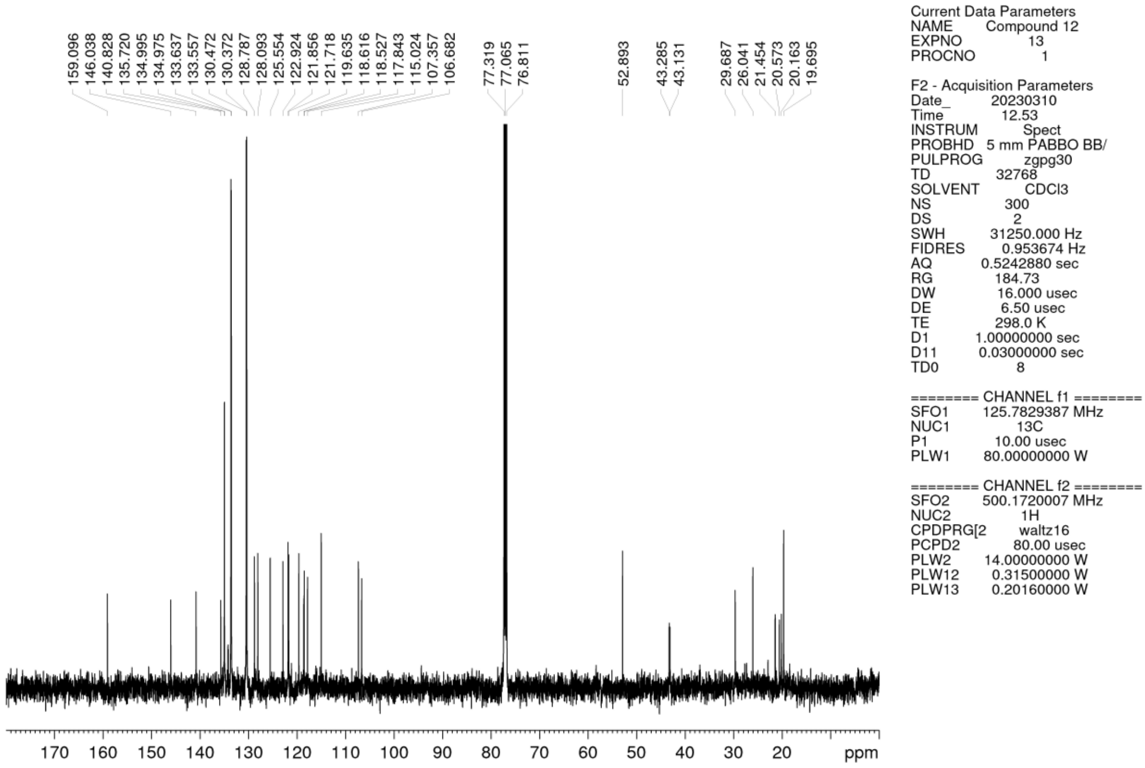

Figure S24. ^{13}C NMR spectra of compound **12** (125 MHz, $CDCl_3$).

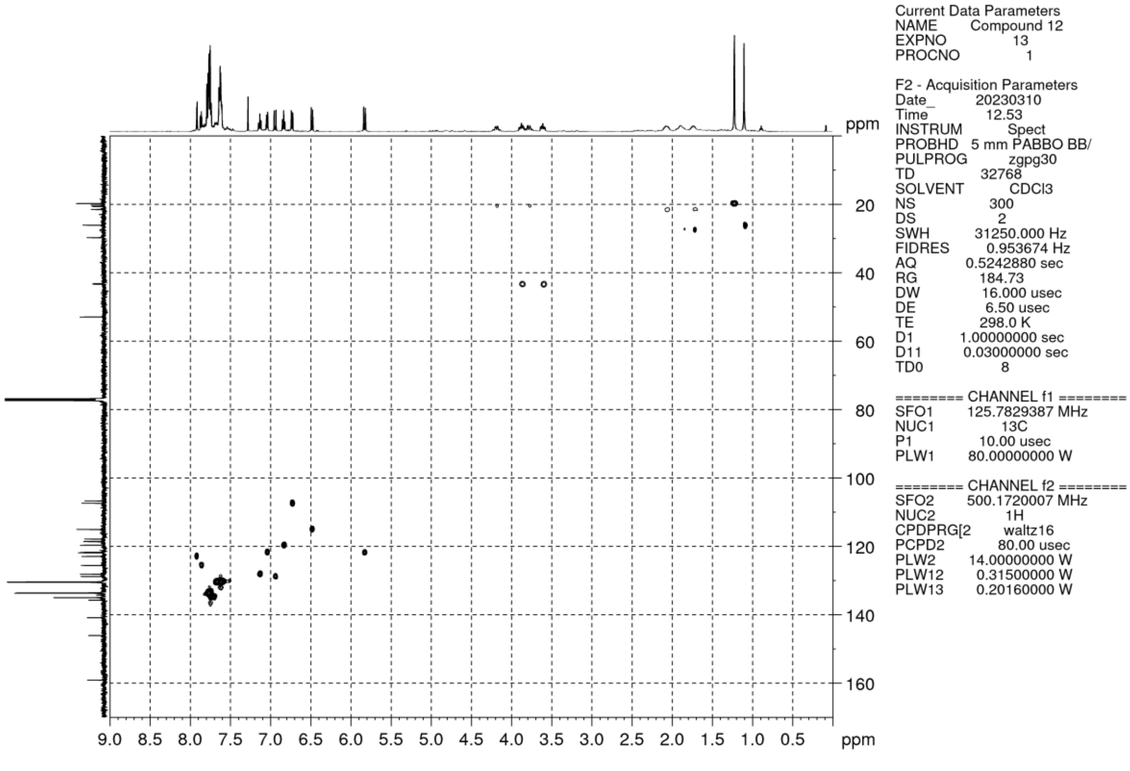

Figure S25. HSQC spectra of compound **12** (500.17 MHz for ^{1}H, 125.78 MHz for ^{13}C, $CDCl_3$).

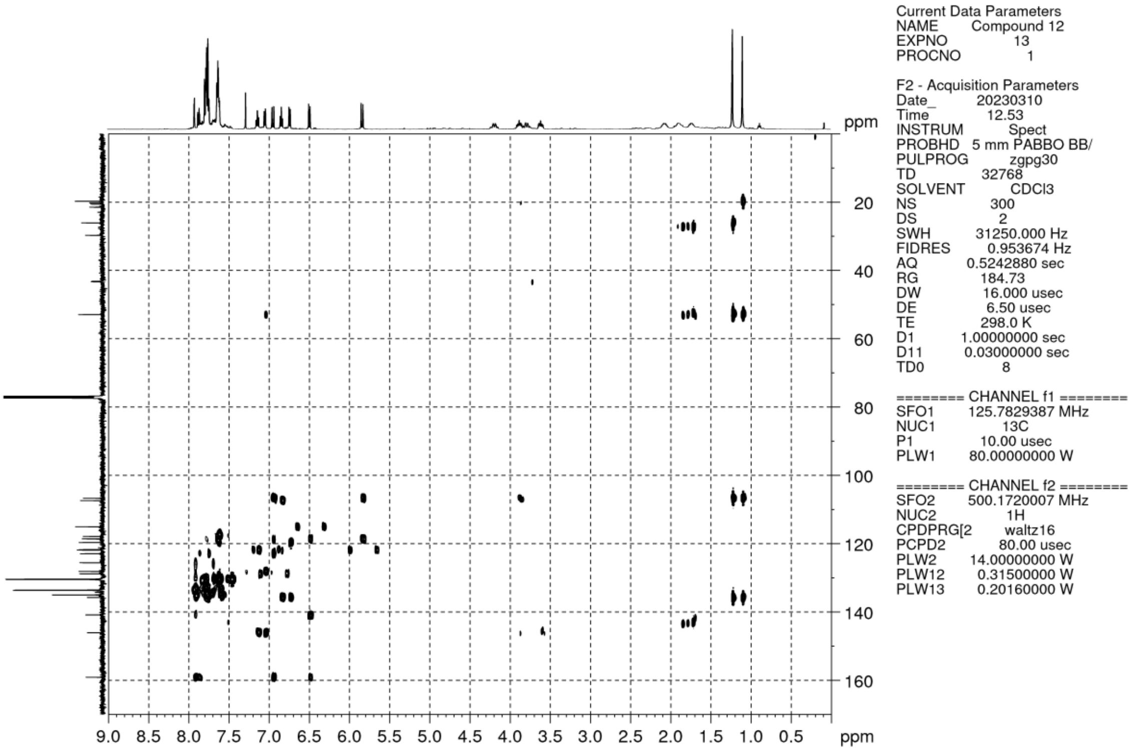

Figure S26. HMBC spectra of compound **12** (500.17 MHz for ^{1}H, 125.78 MHz for ^{13}C, $CDCl_3$).

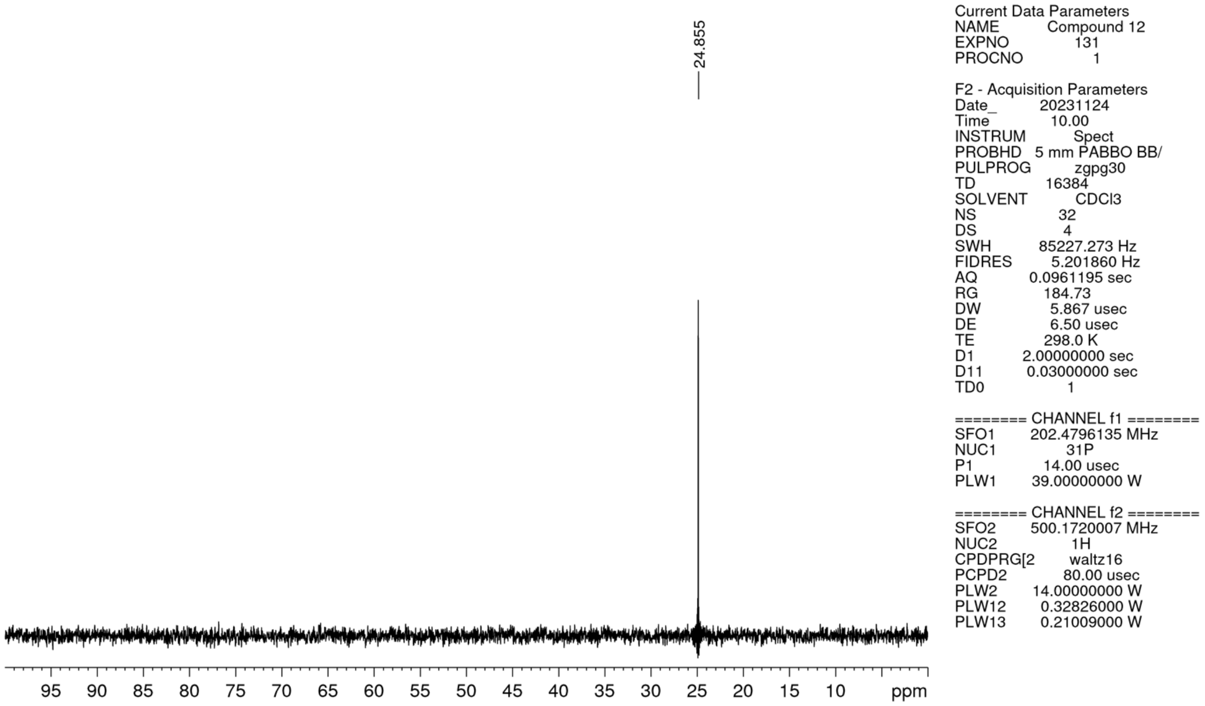

Figure S27. ^{31}P NMR spectra of compound **12** (202 MHz, $CDCl_3$).

Compound 13: ^{1}H NMR (500 MHz, $CDCl_3$): δ = 1.01 (s, 3H), 1.21 (s, 3H), 1.68 (m, 2H), 2.04 (m, 2H), 3.12 (m, 1H), 3.22 (m, 1H), 3.80 (m, 1H), 4.03 (m, 1H), 5.82 (d, *J* = 10.3 Hz, 1H), 6.45

(d, J = 7.8 Hz, 1H), 6.60 (d, J = 9.4 Hz, 1H), 6.84 (t, J = 7.4 Hz, 1H), 6.89 (d, J = 10.4 Hz, 1H), 7.04 (d, J = 7.2 Hz, 1H), 7.11 (t, J = 7.6 Hz, 1H), 7.65 (m, 6H), 7.77 (m, 11H), 7.94 (m, 3H) ppm. ^{13}C NMR (125 MHz, $CDCl_3$): δ = 19.81, 20.59, 22.37, 22.77, 25.95, 29.75, 29.87, 42.94, 52.25, 106.65, 106.67, 115.29, 117.90, 118.52, 118.61, 119.44, 121.68, 122.11, 122.74, 125.68, 127.73, 128.26, 130.41, 130.51, 133.64, 133.72, 134.99, 135.89, 140.86, 146.97, 159.54 ppm.

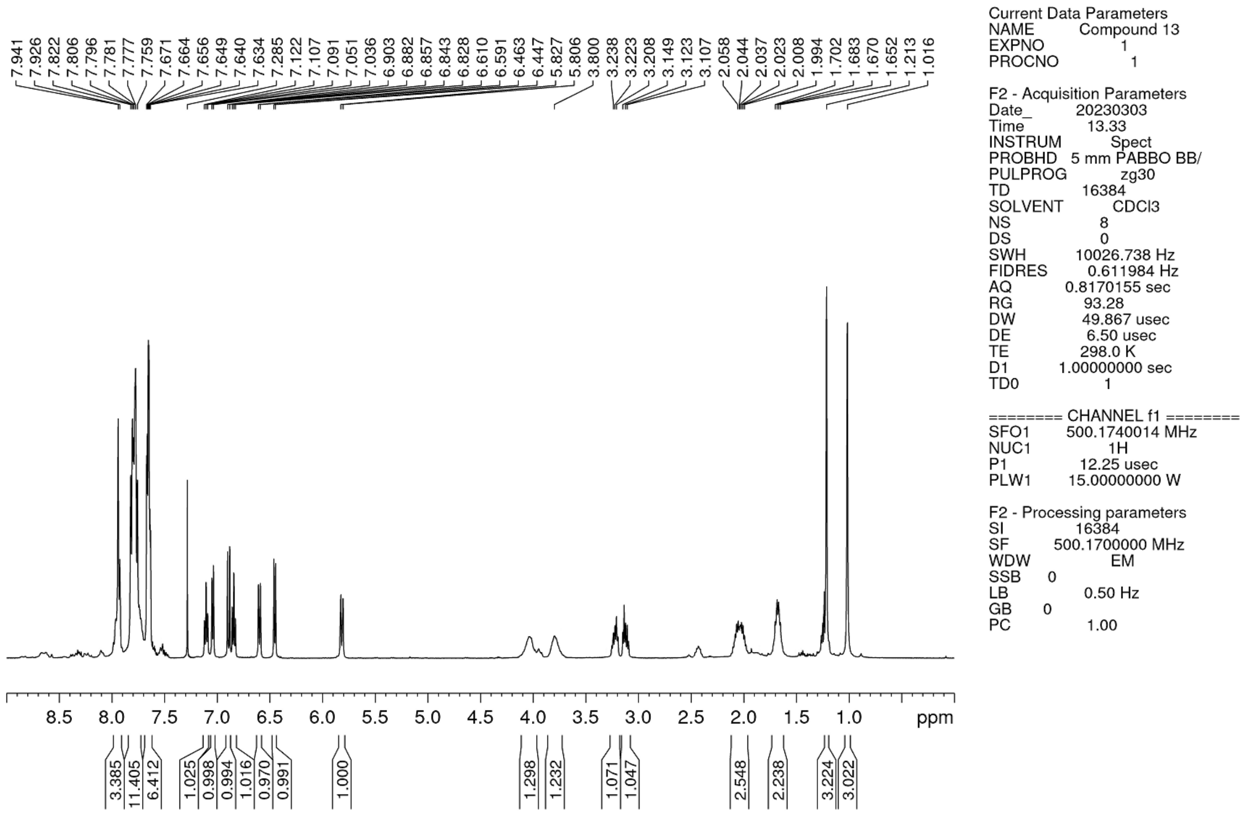

Figure S28. ^{1}H NMR spectra of compound **13** (500 MHz, $CDCl_3$).

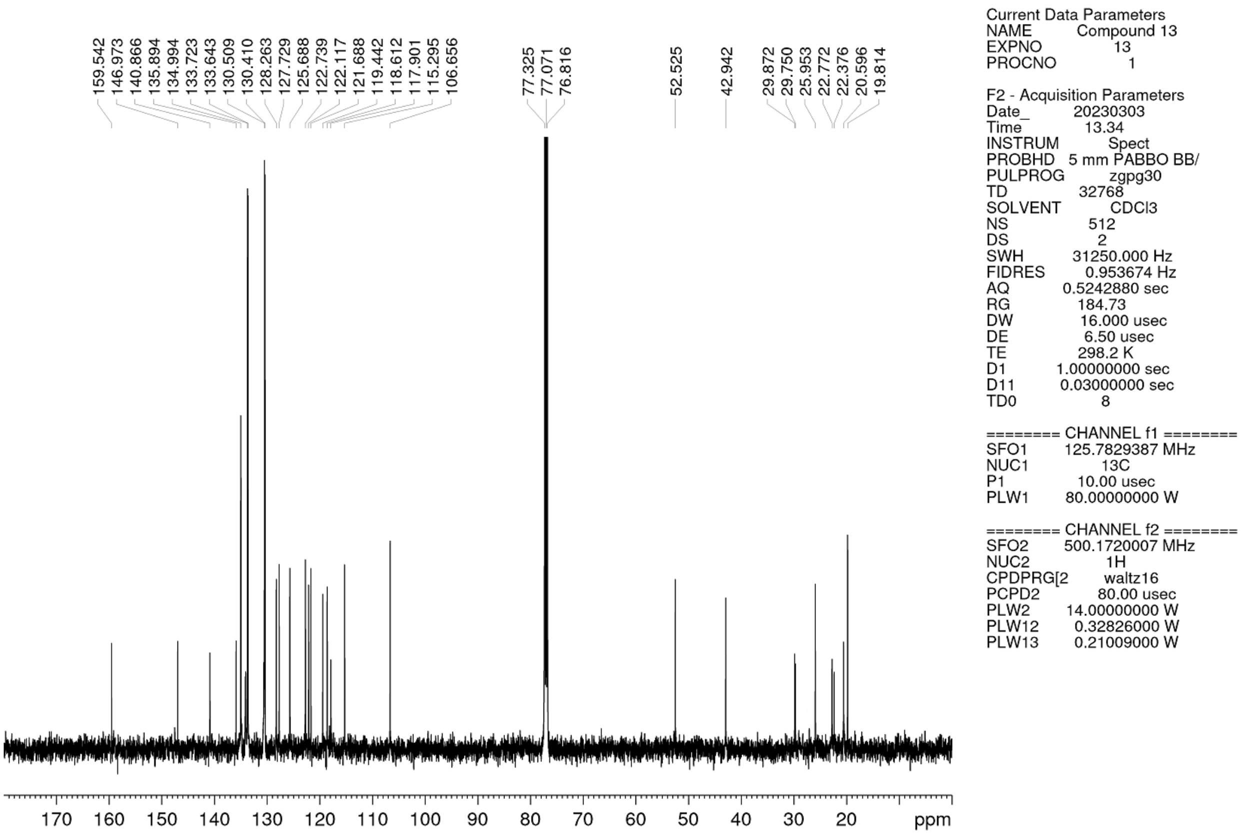

Figure S29. ^{13}C NMR spectra of compound **13** (125 MHz, $CDCl_3$).

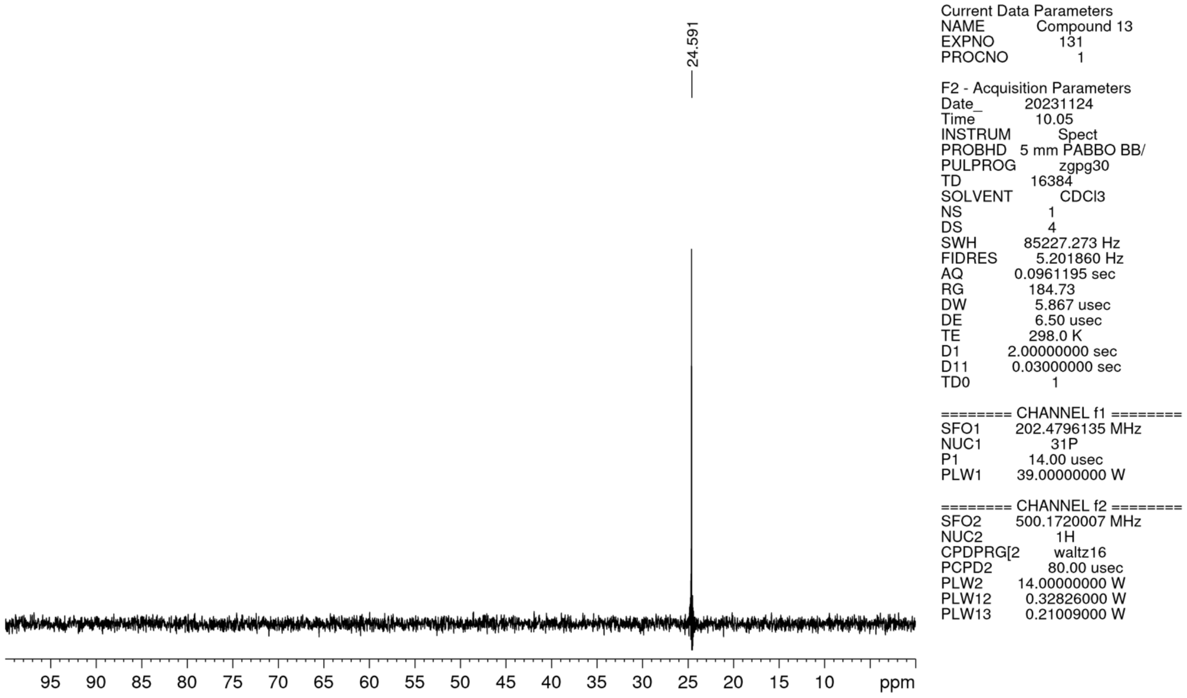

Figure S30. ^{31}P NMR spectra of compound **13** (202 MHz, $CDCl_3$).

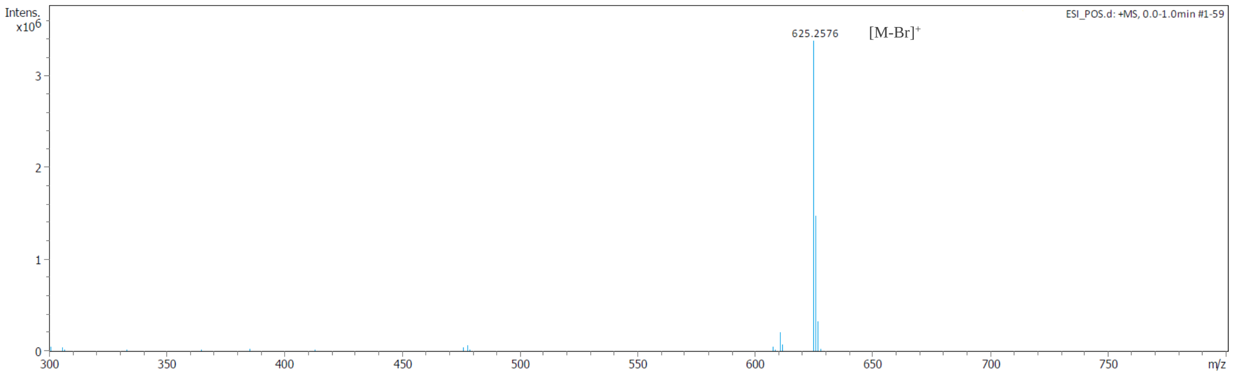

Figure S31. ESI-HRMS spectra of compound **13**.

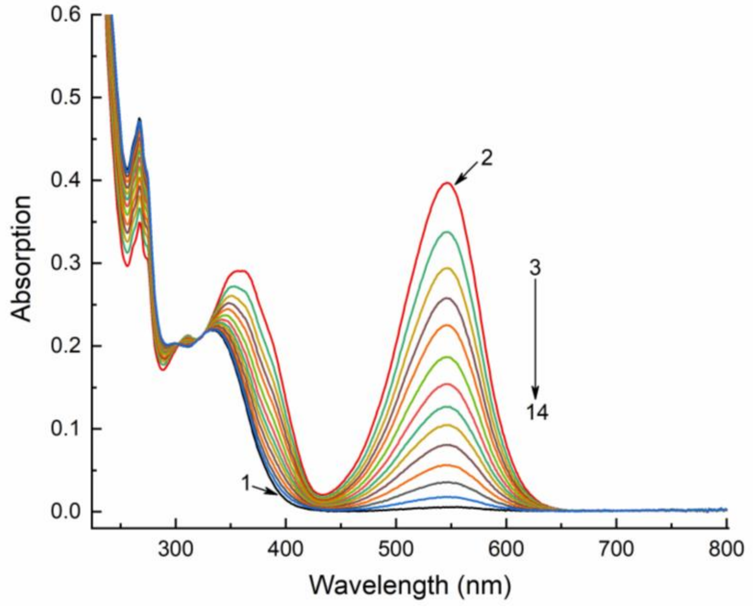

Figure S32. Absorption spectra of **13** in ethanol in spiropyran (1) and merocyanine forms (2-14) measured before (1) and upon UV-irradiation (2) through a UFS-1 light filter and during bleaching in the dark (3-14). C = 10^{-4} M, l = 0.1 cm.

Compound 14: ^{1}H NMR (500 MHz, $CDCl_3$): δ = 1.12 (s, 3H), 1.21 (s, 3H), 1.52 (m, 3H), 1.71 (m, 3H), 3.11 (m, 2H), 3.67 (m, 1H), 3.88 (m, 1H), 5.96 (d, *J* = 10.4 Hz, 1H), 6.45 (d, *J* = 7.7 Hz, 1H), 6.64 (d, *J* = 9.1 Hz, 1H), 6.81 (t, *J* = 7.4 Hz, 1H), 6.92 (d, *J* = 9.1 Hz, 1H), 7.03 (d, *J* = 7.1 Hz, 1H), 7.11 (t, *J* = 7.6 Hz, 1H), 7.67 (m, 7H), 7.78 (m, 10H), 7.90 (m, 2H) ppm. ^{13}C NMR (125 MHz, $CDCl_3$): δ = 19.79, 20.55, 22.70, 22.73, 22.95, 26.14, 28.11, 28.24, 28.65, 43.32, 52.55, 106.62, 106.87, 115.32, 117.88, 118.56, 118.68, 119.31, 121.64, 122.29, 122.73, 125.57, 127.65, 128.32, 130.45, 130.55, 133.57, 133.65, 135.03, 135.05, 136.05, 140.71, 146.99, 159.65 ppm.

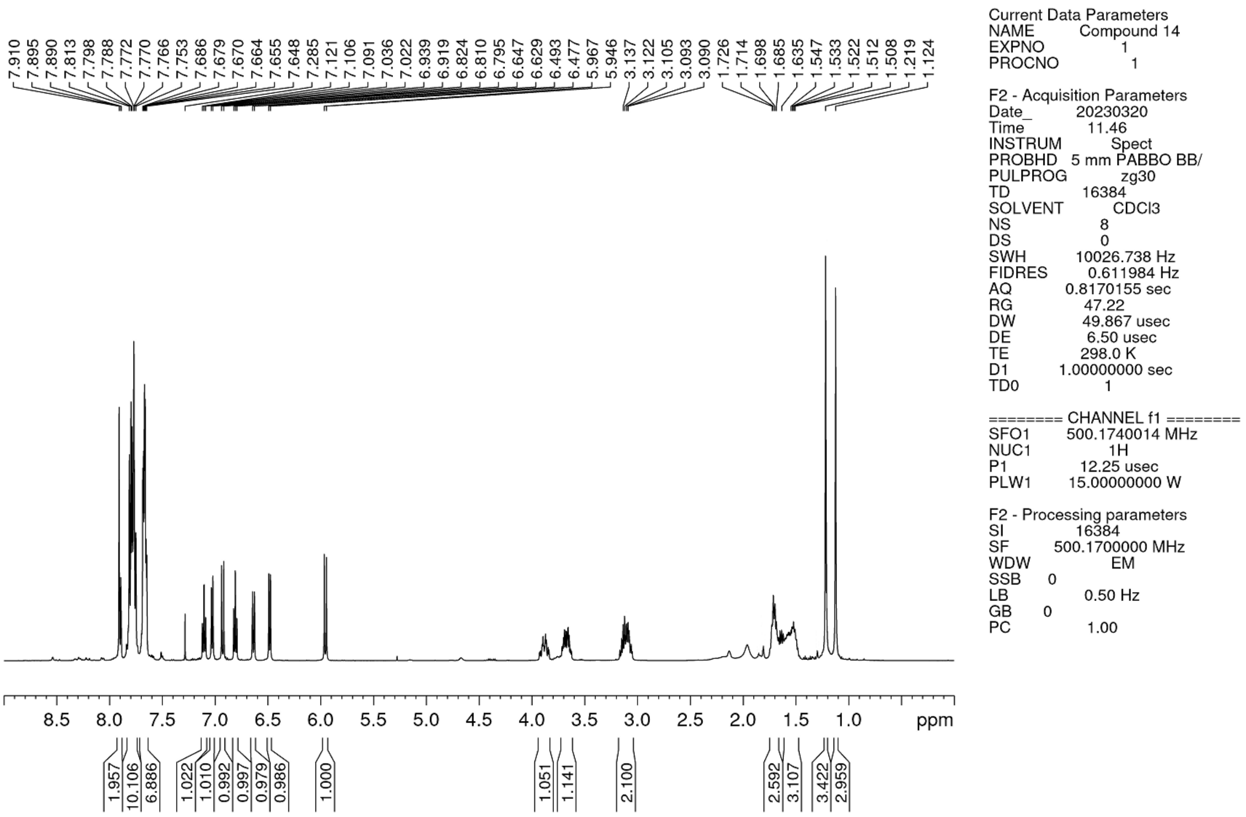

Figure S33. ^{1}H NMR spectra of compound **14** (500 MHz, $CDCl_3$).

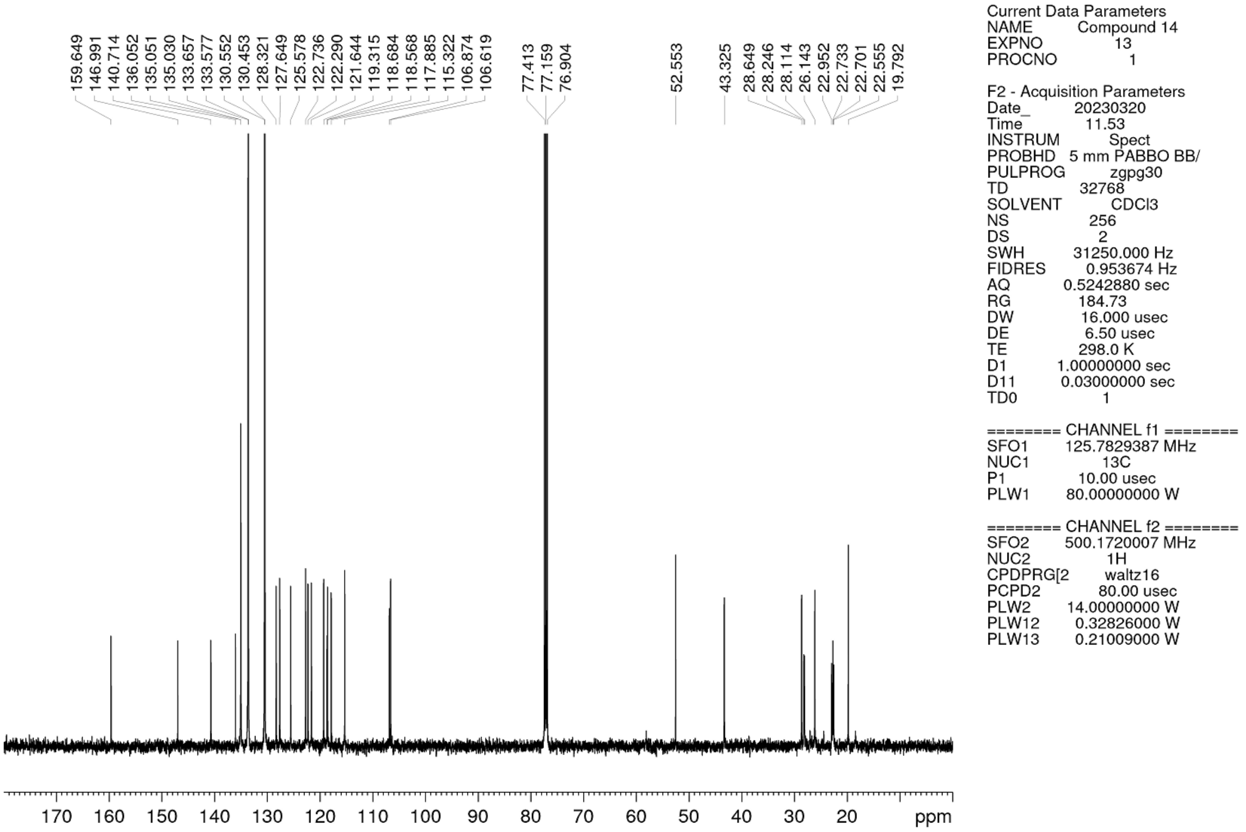

Figure S34. ^{13}C NMR spectra of compound **14** (125 MHz, $CDCl_3$).

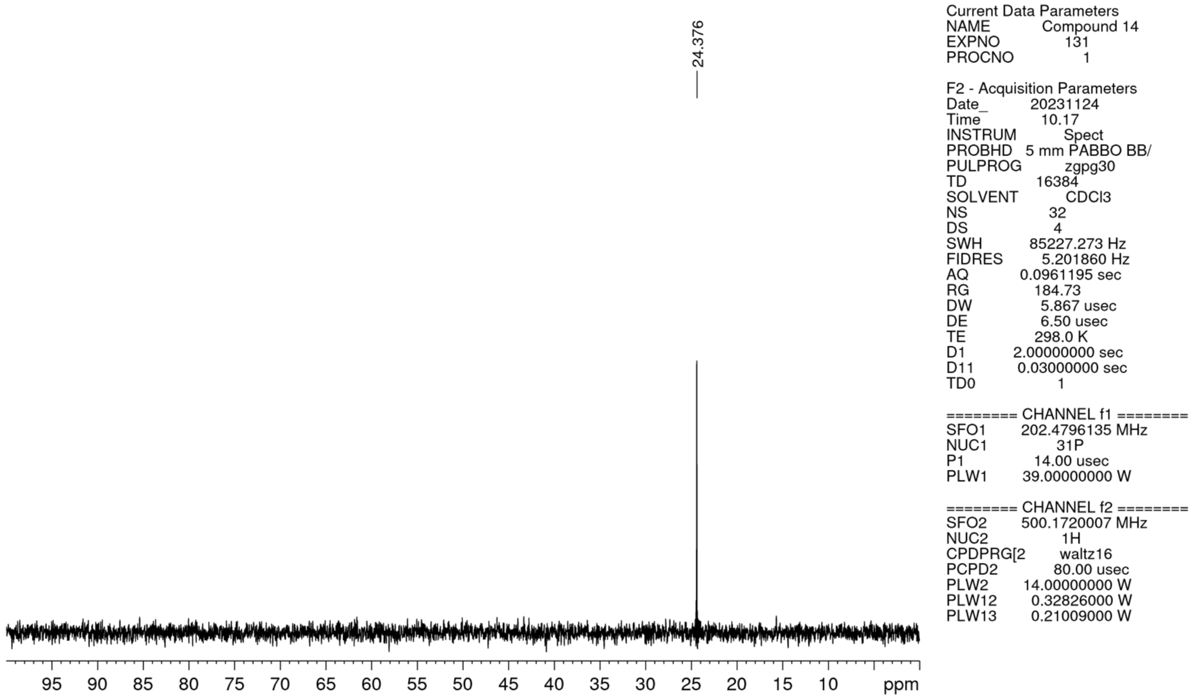

Figure S35. ^{31}P NMR spectra of compound **14** (202 MHz, $CDCl_3$).

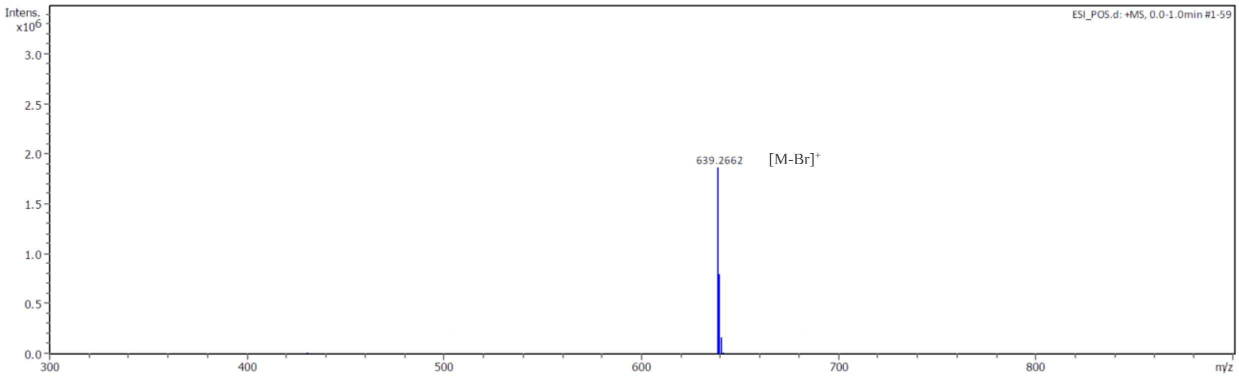

Figure S36. ESI-HRMS spectra of compound **14**.

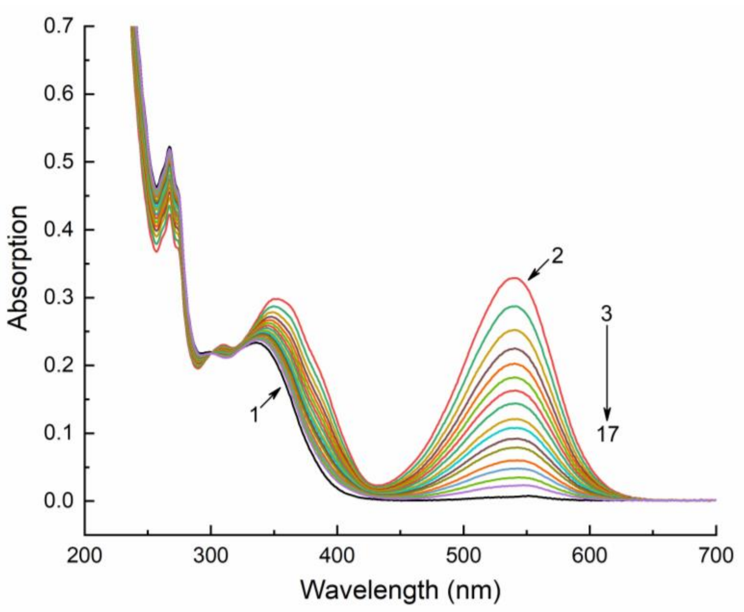

Figure S37. Absorption spectra of **14** in ethanol in spiropyran (1) and merocyanine forms (2-17) measured before (1) and upon UV-irradiation (2) through a UFS-1 light filter and during bleaching in the dark (3-17). C = 10^{-4} M, l = 0.1 cm.

Compound 15: ^{1}H NMR (500 MHz, $CDCl_3$): δ = 1.14 (s, 3H), 1.25 (s, 3H), 1.48 (m, 2H), 1.63 (m, 7H), 1.98 (m, 2H), 3.10 (m, 2H), 3.74 (m, 1H), 3.86 (m, 1H), 5.86 (d, *J* = 10.4 Hz, 1H), 6.51 (d, *J* = 7.7 Hz, 1H), 6.68 (d, *J* = 9.0 Hz, 1H), 6.83 (t, *J* = 7.4 Hz, 1H), 6.94 (d, *J* = 10.4 Hz, 1H), 7.06 (d, *J* = 7.2 Hz, 1H), 7.13 (t, *J* = 7.6 Hz, 1H), 7.69 (m, 8H), 7.81 (m, 11H), 7.94 (dd, J_1 = 8.9, J_2 = 2.7 Hz, 1H), 7.98 (d, *J* = 2.7 Hz, 1H) ppm. ^{13}C NMR (125 MHz, $CDCl_3$): δ = 19.79, 22.54, 22.65, 22.93, 26.06, 26.93, 28.72, 30.21, 30.34, 43.49, 52.59, 106.64, 106.77, 115.41, 118.01, 118.62, 118.69, 119.21, 121.61, 122.07, 122.75, 125.69, 127.71, 130.42, 130.52, 133.63, 133.71, 135.00, 135.92, 136.05, 140.77, 147.04, 159.71 ppm.

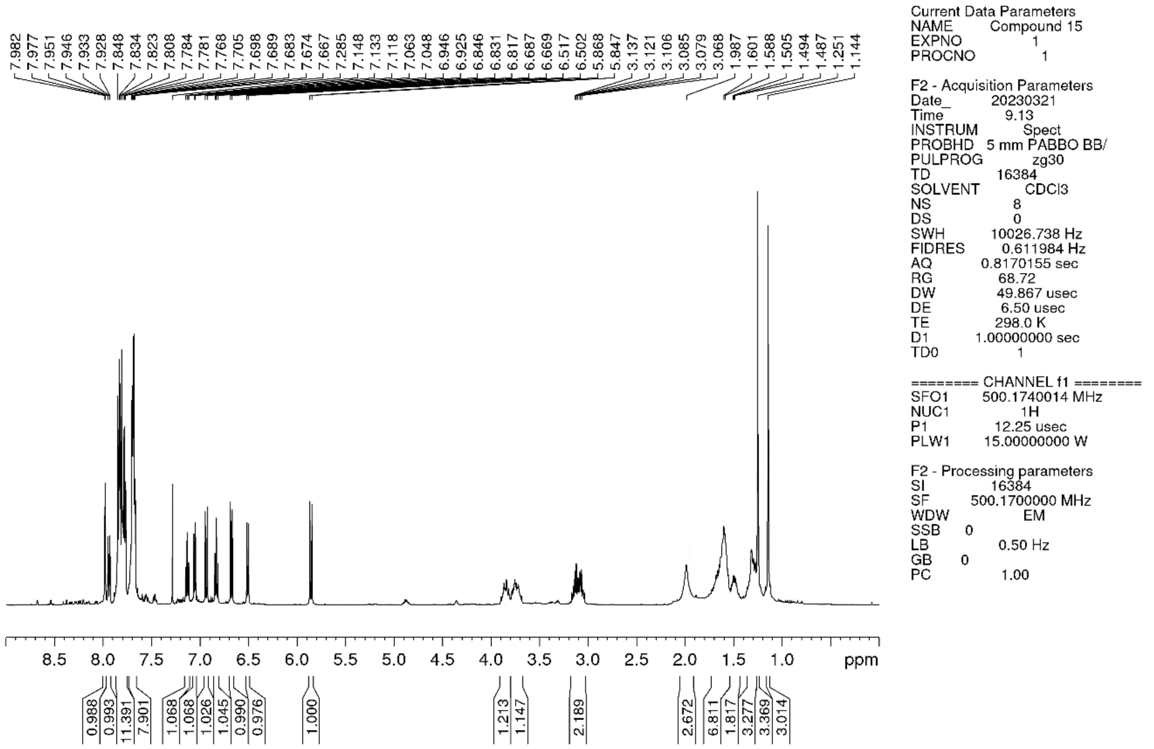

Figure S38. ^{1}H NMR spectra of compound **15** (500 MHz, $CDCl_3$).

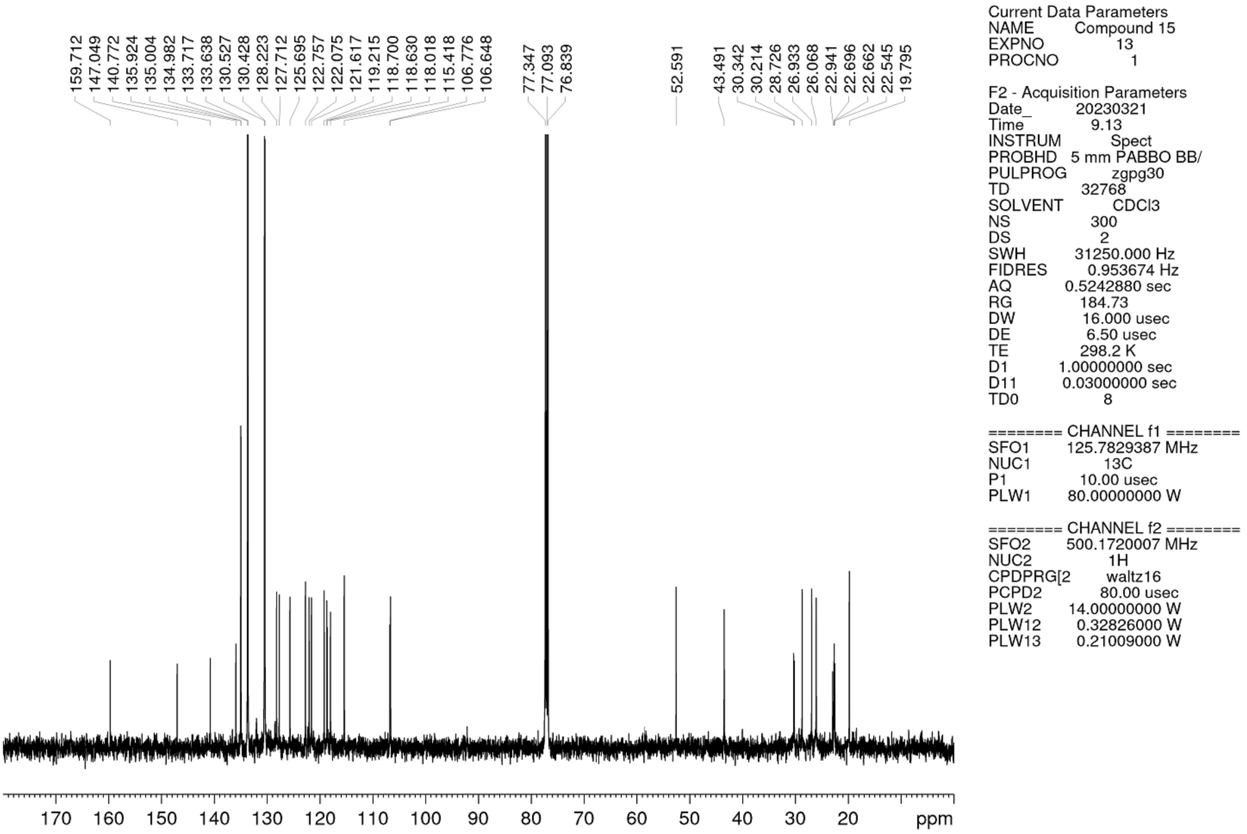

Figure S39. ^{13}C NMR spectra of compound **15** (125 MHz, $CDCl_3$).

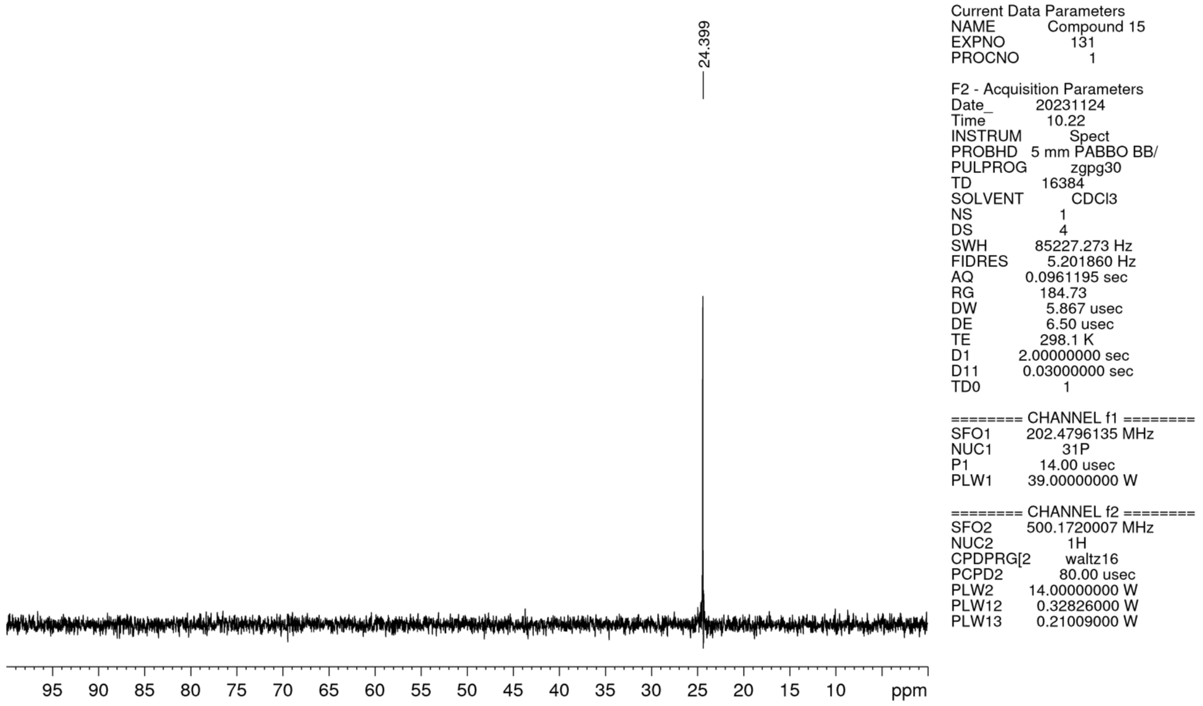

Figure S40. ^{31}P NMR spectra of compound **15** (202 MHz, $CDCl_3$).

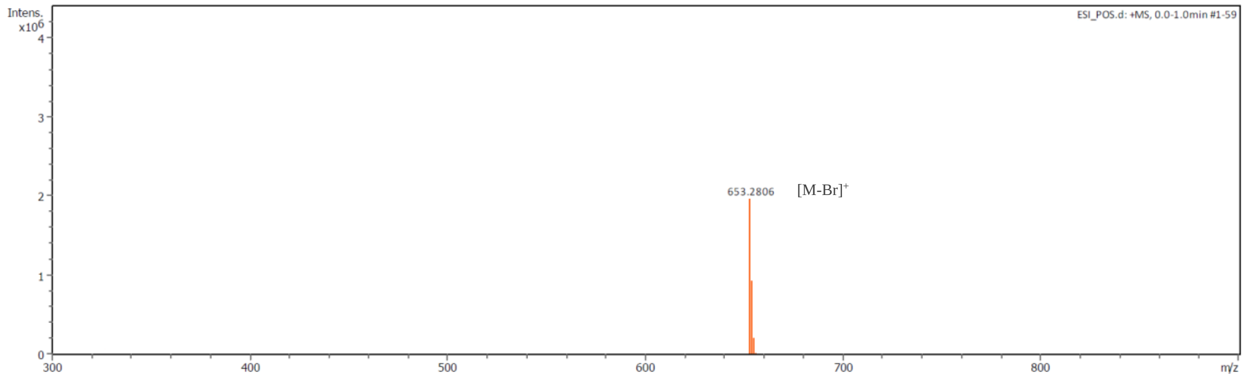

Figure S41. ESI-HRMS spectra of compound **15**.

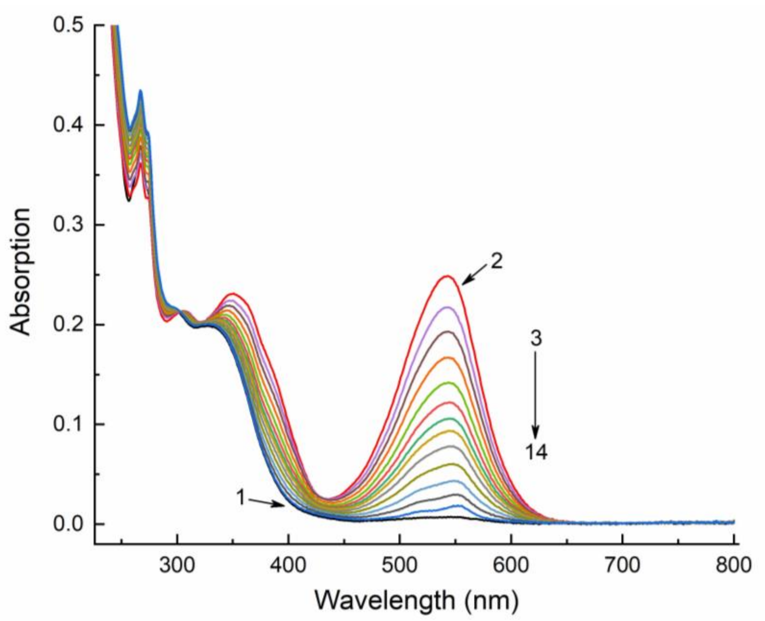

Figure S42. Absorption spectra of **15** in ethanol in spiropyran (1) and merocyanine forms (2-14) measured before (1) and upon UV-irradiation (2) through a UFS-1 light filter and during bleaching in the dark (3-14). C = 10^{-4} M, l = 0.1 cm.

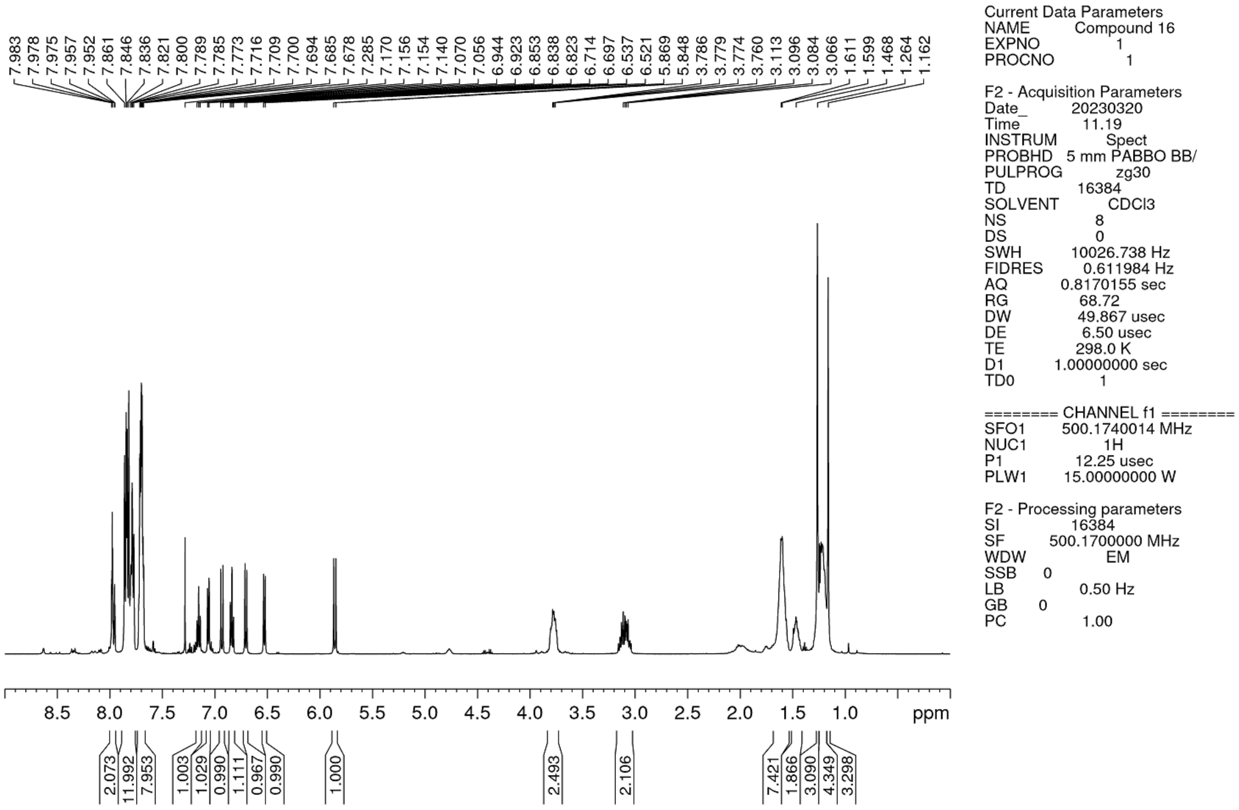

Figure S43. ^{1}H NMR spectra of compound **16** (500 MHz, $CDCl_3$).

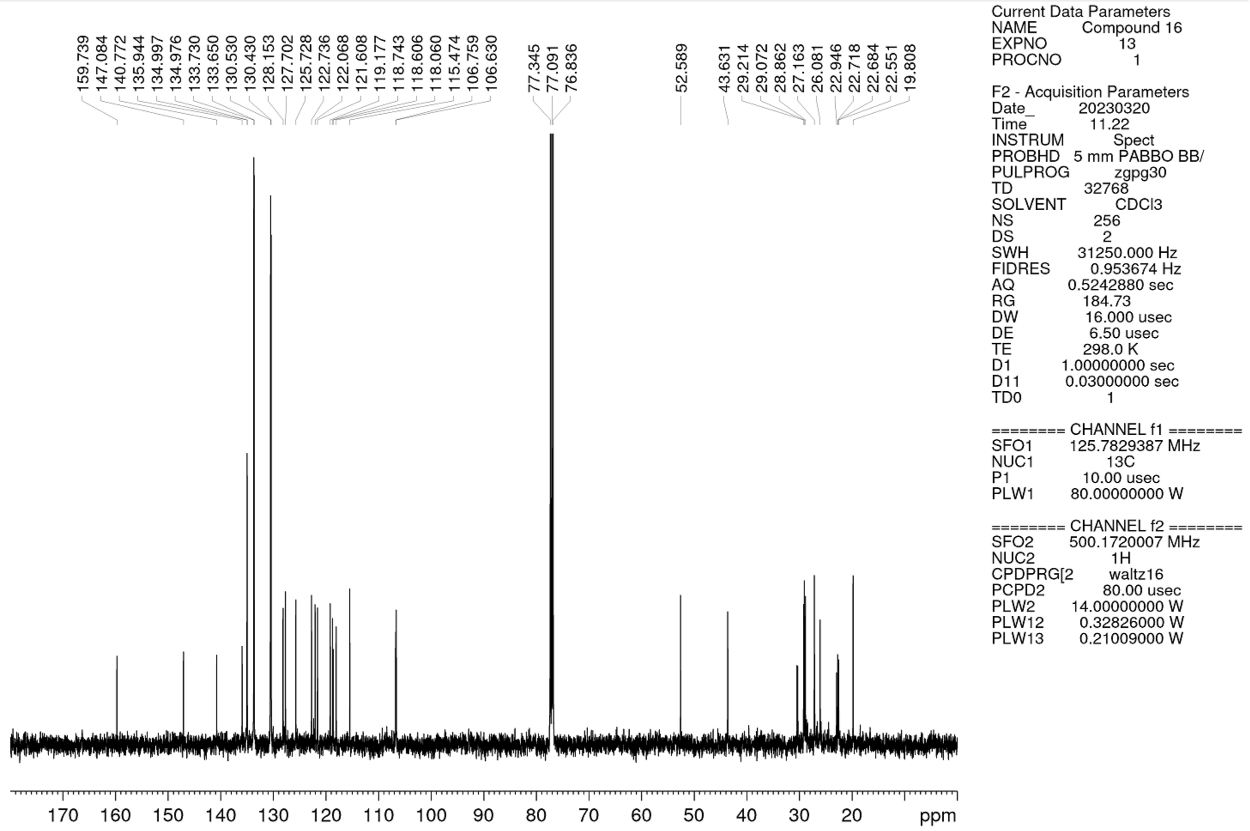

Figure S44. ^{13}C NMR spectra of compound **16** (125 MHz, $CDCl_3$).

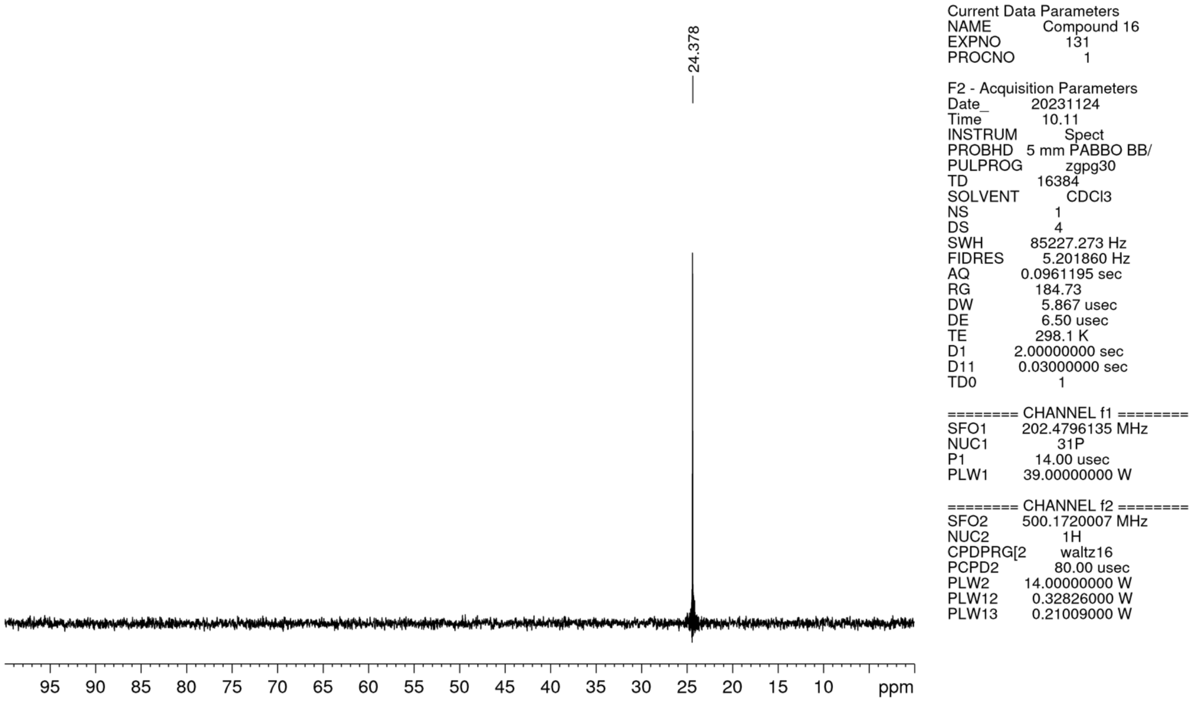

Figure S45. ^{31}P NMR spectra of compound **16** (202 MHz, $CDCl_3$).

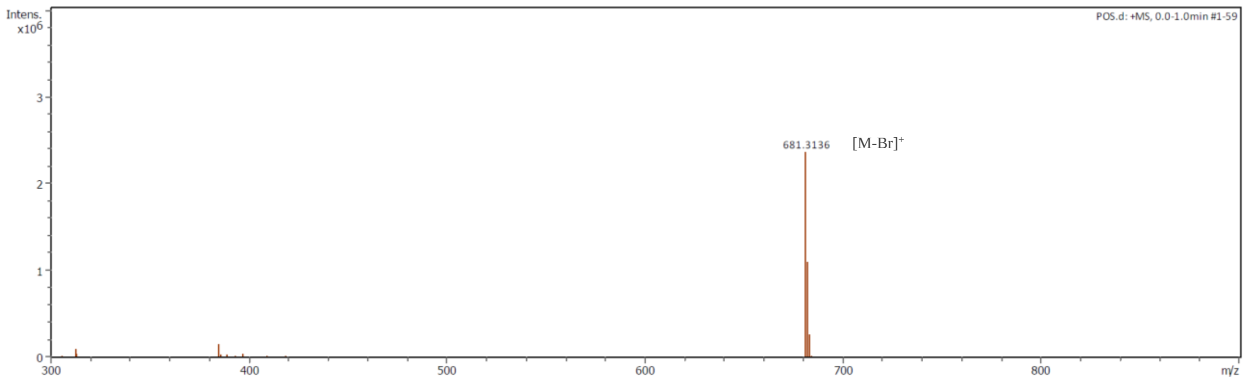

Figure S46. ESI-HRMS spectra of compound **16**.

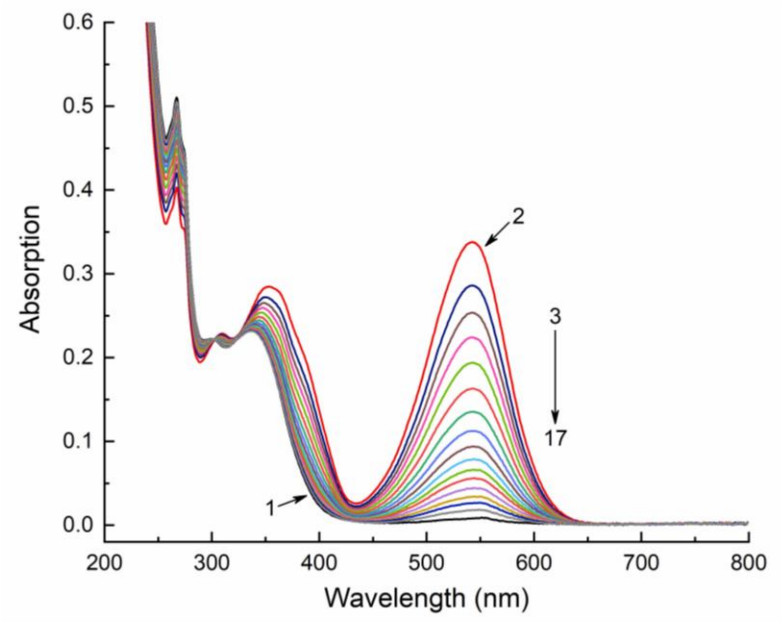

Figure S47. Absorption spectra of **16** in ethanol in spiropyran (1) and merocyanine forms (2-17) measured before (1) and upon UV-irradiation (2) through a UFS-1 light filter and during bleaching in the dark (3-17). C = 10^{-4} M, l = 0.1 cm.
